# On “Decisions and Revisions Which a Minute Will Reverse”: Consciousness, The Unconscious and Mathematical Modeling of Thinking

**DOI:** 10.3390/e23081026

**Published:** 2021-08-09

**Authors:** Arkady Plotnitsky

**Affiliations:** Literature, Theory and Cultural Studies Program, Philosophy and Literature Program, Purdue University, West Lafayette, IN 47907, USA; plotnits@purdue.edu

**Keywords:** quantum-like theories and models, consciousness, the unconscious, reality, reality without realism, quantum individuality

## Abstract

This article considers a partly philosophical question: What are the ontological and epistemological reasons for using quantum-like models or theories (models and theories based on the mathematical formalism of quantum theory) vs. classical-like ones (based on the mathematics of classical physics), in considering human thinking and decision making? This question is only partly philosophical because it also concerns the scientific understanding of the phenomena considered by the theories that use mathematical models of either type, just as in physics itself, where this question also arises as a physical question. This is because this question is in effect: What are the physical reasons for using, even if not requiring, these types of theories in considering quantum phenomena, which these theories predict fully in accord with the experiment? This is clearly also a physical, rather than only philosophical, question and so is, accordingly, the question of whether one needs classical-like or quantum-like theories or both (just as in physics we use both classical and quantum theories) in considering human thinking in psychology and related fields, such as decision science. It comes as no surprise that many of these reasons are parallel to those that are responsible for the use of QM and QFT in the case of quantum phenomena. Still, the corresponding situations should be understood and justified in terms of the phenomena considered, phenomena defined by human thinking, because there are important differences between these phenomena and quantum phenomena, which this article aims to address. In order to do so, this article will first consider quantum phenomena and quantum theory, before turning to human thinking and decision making, in addressing which it will also discuss two recent quantum-like approaches to human thinking, that by M. G. D’Ariano and F. Faggin and that by A. Khrennikov. Both approaches are ontological in the sense of offering representations, different in character in each approach, of human thinking by the formalism of quantum theory. Whether such a representation, as opposed to only predicting the outcomes of relevant experiments, is possible either in quantum theory or in quantum-like theories of human thinking is one of the questions addressed in this article. The philosophical position adopted in it is that it may not be possible to make this assumption, which, however, is not the same as saying that it is impossible. I designate this view as the reality-without-realism, RWR, view and in considering strictly mental processes as the ideality-without-idealism, IWI, view, in the second case in part following, but also moving beyond, I. Kant’s philosophy.

Do I dare

Disturb the universe?

In a minute there is time

For decisions and revisions which a minute will reverse.

—T. S. Eliot, “The Love Song of J. Alfred Prufrock” (ll. 45–46)

## 1. Introduction

The history of mathematical modeling outside physics, including when dealing with human thinking and behavior, has been dominated by the use of classical-like models, especially probabilistic or statistical ones, borrowed from classical statistical physics or chaos and complexity theories. During the last few decades, however, quantum-like models, models based in the mathematical formalism of quantum theory, became more current in biology, neuroscience, psychology, decision science and economics. Beginning with A. Tversky and D. Kahneman’s pioneering work in the 1970–80s (e.g., [1,2]), it has been primarily the presence of probabilistic data akin to those of quantum physics that suggested using quantum-like models (e.g., [3]). For brevity, I adopt abbreviations Q-like and C-like models or theories, in the spirit of P. Dirac’s terms of q-numbers and c-numbers in quantum theory. In specifying the corresponding Q-like models or theories, I shall use QM-like and QFT-like. (Alternative theories of quantum phenomena, such as Bohmian mechanics, will not be considered here, beyond a few brief mentions, by way of contrast to the standard versions of QM and QFT.) I shall properly discuss the distinction between models and theories in Section 3. Briefly, the model adopted by a theory refers here to its mathematical formalism, while a theory refers to the overall assemblage of concepts comprising it, not all of which are mathematical.

The aim of this article is to consider a partly philosophical question: What are the ontological and epistemological reasons for using Q-like models or theories, vs. C-like ones, in considering human thinking and decision making? This question is only partly philosophical, because it also concerns the scientific understanding of the phenomena considered by the theories that use mathematical models of either type, just as in physics where this question also arises as a physical question. This is especially the case in quantum theory, QM or QFT, and its interpretations, on which its physics and mathematics depend. While this dependence is also found in classical physics and relativity, in QM and QFT the question of interpretation is particularly pressing, given a different nature of the formalism of these theories and how this formalism relates to the experimental data considered. This difference is due to such features as the apparently unavoidable role of complex numbers, the noncommutative nature of the key mathematical operations involved and the use of rules, such as Born’s rule, added to the formalism, through which one relates to the probabilities of the outcomes of quantum experiments. Such rules are necessary because these probabilities and these outcomes themselves are real numbers (in the case of outcomes, technically, rational numbers). No other than probabilistic predictions are possible in quantum physics because identically prepared quantum experiments lead to different outcomes, in contrast with in classical physics and relativity, where ideally exact predictions are possible in dealing with individual or small systems. The question, then, is: What are the physical reasons for using, even if not requiring, these types of theories in considering quantum phenomena, which these theories predict fully in accord with the experiment? This is clearly also a physical, rather than only philosophical, question. So is, accordingly, the question of whether one needs C-like or Q-like theories or both (just as in physics we use both classical and quantum theories) in considering human thinking in psychology and related fields, such as decision science.

C-like or Q-like models need not, and here will not, be seen as arising from the physics of the brain as a neurological system by assuming that the physical processes responsible for our thinking are classical or quantum, respectively. Even though most neurological theories assumed that the physics of the brain responsible for our thinking is classical, there are (hypothetical) theories that assume consciousness or thinking in general to be an effect of the quantum physics of the brain, such as, prominently, those by R. Penrose, beginning with [4]. How the physics of the brain makes thinking or consciousness, as we experience it, possible remains an unanswered question, sometimes referred to as “the hard problem of consciousness” [5]. It appears to be designated as the hard problem of consciousness, rather than thinking, because our manifested experience is that of consciousness, and not that of the unconscious thinking, inferred from our conscious thinking, an inference sometimes denied. In any event, such theories, also known as “physicalist”, will be (mostly) put aside here. The physical constitution of the brain will be decoupled from mental processes, for which it is, however, assumed to be responsible here. The brain will be treated, in terms of information theory, as, *physically*, a “black box”, responsible for information, by relating the informational input and output, as encountered from both the inside and the outside of a human subject. This move was pointedly made by S. Freud (who started his scientific career as a neuroscientist and initially aimed to approach thinking and memory neurologically) in establishing psychoanalysis, pursued by him as a scientific project, as a science of the mind, responsible for, but analytically decoupled from, the functioning of the brain (e.g., [6,7]). Freud did not speak in informational terms, which entered scientific discourse, including in the biological sciences, with the rise of information theory in the late 1940s, due primarily to C. E. Shannon (although there were earlier anticipations, even during the lifetime of Freud, who died in 1938). It is a more complex question, addressed in Section 4, whether the mind is a *mental* black box in the sense of the possibility of accounting for how the mind, for example, as an informational system of the Q-like type, produces such outputs. While decoupling the mind from the brain, Freud aimed at accounting, through the unconscious, for the workings of the mind, which a psychological, for example, psychoanalytic, theory could represent, thus providing a mental ontology of these workings. Some Q-like approaches to be considered here make this type of assumption as well, although the mental ontologies they consider are different from that of Freud, given that they are represented by the formalism of quantum theory and thus mathematically. Mathematics played no role in Freud’s representation of human thinking, a representation that was conceptual and narrative, with that of the Oedipal complex as the most famous and most controversial narrative of Freud’s psychoanalysis. Whether such a representation, mathematical or conceptual (or narrative), is possible is one of the questions asked by this article, which does not make the assumption that it is. The absence of this assumption defined the philosophical position of this article, the position that I shall designate as the reality-without-realism, RWR, view or when referring to strictly mental processes as the ideality-without-idealism, IWI, view, in the latter case in part following, but also moving beyond, I. Kant’s philosophy. It is important that this position only assumes that it may not be possible to make this assumption, which is not the same as saying that it is impossible.

But why are such considerations important in the first place? One could justify the use of Q-like theories or models because they appear to work better than C-like ones, even though this view might be and has been challenged. Such debates are, however, not the same as refutations of one or another approach. In addition, Q-like models are already available rather than having to be found to adequately deal with the phenomena considered, as it was in the case of quantum physics, which requires one to invent QM and then QFT (first in low and then high-energy regimes) to account for quantum phenomena. One could also disregard the question of how quantum phenomena or our predictions concerning them are possible or why it may not be possible to answer this question, in short, the question of interpretation, in QM or QFT. The practice of theoretical physics is possible and can be effective apart from such considerations. I would argue, however, that understanding the reasons grounding this use allow us to make important conclusions about the nature of the phenomena considered by these theories and these theories themselves. To put it in strong terms, finding such reasons, even if the theory or model considered is already available, is important because otherwise we do not have a rigorous theory or a rigorous model. These considerations remain relevant in quantum theory or fundamental physics in general (physics that deal with the ultimate constitution of nature). In fact, even more is at stake in such considerations in physics and beyond. In physics, they may help us to solve outstanding problems of fundamental physics, such as those of QFT (which still has such problems) or those that prevent us from developing a viable theory of quantum gravity. There is no such a theory thus far, at least, not a generally accepted one. It is not inconceivable either that we will need to move beyond those Q-like theories and models that we know use, such as QM- and QFT-like ones (perhaps we will use quantum-gravity-like models, once they become available), or even beyond Q-like theories and models in general, without, however, restricting ourselves to C-like ones, beyond physics. Indeed, a recent use of quantum-informational-like theories and models, to be discussed in this article, is itself this type of change, admittedly, still within Q-like paradigm, but a change nevertheless, with some radical implications.

The aim of this paper, then, is to address some of the reasons why Q-like theories or models are possible or even necessary beyond quantum physics, in particular in dealing with human thinking in psychology and decision sciences. It should come as no surprise that many of these reasons are parallel to those that are responsible for the use of QM and QFT in the case of quantum phenomena. Still, the corresponding situations should be understood and justified in terms of the phenomena considered, phenomena defined by human thinking. This is also necessary because there are important differences between these phenomena and quantum phenomena, which this article aims to address. In order to do so, however, I shall, first, in the next two sections, Section 2 and Section 3, consider quantum phenomena and quantum theory. Section 4 and the Conclusion address human thinking and decision making. 

## 2. Quantum Phenomena and Quantum Theory: Physical Postulates and Mathematical Formalism

The aim on this section is to consider why quantum theory, QM or QFT, has a particular physical nature and mathematical structure, and why it allows for the type of interpretations to be considered here under the heading of “reality without realism” (RWR), one of which will be adopted by this article. Do these phenomena require these types of theories or these types of interpretations of these theories, rather than merely allowing for them? It would be difficult to argue such a case, and it is not my aim to do so. 

For nearly a century now, since the publication of J. von Neumann’s *The Mathematical Foundation of Quantum Mechanics* [8], QM (and subsequently QFT) most commonly use as their mathematical formalism the Hilbert-space formalism. There are other versions, some more abstract ones, such as those of C*-algebras and, more recently, sheaf theory and category theory, all of which are more or less equivalent mathematically. The Hilbert-space formalism remains dominant, however. I outline the key features of this formalism and its use in QM or QFT:

(1) A Hilbert-space, H, over complex numbers, ℂ, used in QM, is an abstract vector space of any dimension, finite or infinite, which possesses the structure of an inner product that allows lengths and angles to be measured, analogously to an n-dimensional Euclidean space (which is a Hilbert space over real numbers, ℝ);
(2) The feature of that formalism, arguably, most crucial for QM and never used in physics previously, is the noncommutativity of the Hilbert-space operators, known as “observables”, which are mathematical entities associated, in terms of probabilistic or statistical predictions, with physically observable quantities by means of (3);
(3) The presence of Born’s rule or an analogous rule (such as von Neumann’s projection postulate or Lüder’s postulate), which establishes the relation between the so-called “quantum amplitudes”, associated with complex Hilbert-space vectors as complex entities, and probabilities as real numbers, by using square moduli or, equivalently, the multiplication of these quantities and their complex conjugates (technically, these amplitudes are first linked to probability densities);
(4) The probabilities involved are nonadditive: the joint probability of two or more mutually exclusive alternatives in which an event might occur is, in general, not equal to the sum of the probabilities for each alternative, as in classical probability theory; instead, it obeys the law of the addition of “amplitudes” for these alternatives, to the sum of which Born’s rule is then applied.

While keeping in mind alternative postulates just mentioned, I shall, for simplicity, refer to Born’s rule from now on. In technical terms, which may be opportune to use here as an illustration how the formalism of QM works and how Born’s rule supplements it, Born’s rule is defined as follows. First, in the case of a self-adjoint Hilbert-space operator *A* (〈Av, w〉=〈v, Aw〉, for all vectors in this space) with a discrete spectrum, Born’s rule states that, if a physical observable associated with *A* is predicted by means of (normalized) wave function $\left|\psi \right.>$, then the outcome, when measured, will be one of the eigenvalues λ of *A*; the probability for that it will correspond to a given eigenvalue $\lambda_{i}$ is equal to $\langle \psi {|P}_{i}|\psi \rangle$, where P_i_ is the projection onto the eigenspace of *A* corresponding to $\lambda_{i}$. If this eigenspace is one-dimensional, spanned by the normalized eigenvector $\left|\lambda_{i}>\right.$, P_i_ = $\left|\lambda_{i}>\right.<\left.\lambda_{i}\right|$ and $\langle \psi {|P}_{i}|\psi \rangle$ = $\langle \psi |\lambda_{i}\rangle \langle \;\lambda_{i}|\psi \rangle$. The probability amplitude is $\langle \lambda_{i}|\psi \rangle$, and Born’s rule means that the corresponding probability is the square of the amplitude or is the amplitude multiplied by its own complex conjugate, or P_i_ = $\langle \lambda_{i}|\psi \rangle^{2}$. If the spectrum has a continuous part, then, by the so-called spectral theorem (which deals with cases when a linear operator or matrix can be diagonalized) tells us that there exists a projection-valued measure *Q*, called the spectral measure of *A*. In this case, the probability that the measurement outcomes lie in a measurable set *M* is $\langle \psi |Q\left(M\right)|\psi \rangle .$ In the simplest case, when ψ is a wave function for a point particle in position space, the probability density function *p (x, y, z)* for predicting a measurement of the position at time *t_1_* equals to ${\left|\psi (x,y,z,t_{1}\right|}^{2}$. 

Even though Born’s or analogous rule are, thus, connected rather naturally mathematically to the formalism of QM, they are, nevertheless, added to this formalism rather than derived from it. We do not know why these postulates work, which makes it tempting to argue that (insofar as it is it allows us to link the formalism to the data) why they work is the greatest mystery of QM. However, then we do not know why the whole scheme works either. There is neither one without the other. There have been attempts to provide reasons or justifications for Born’s rule from (presumably) more basic principles or postulates, rather than seeing it as a primary postulate. I shall put these attempts aside, because, while they are conceptually valid, none of them (at least among those commonly accepted) derives Born’s rule from the formalism of QM. It is still a postulate, even if derived from other postulates assumed to be more basic. 

The mathematical features just outlined were not initially assumed, but, beginning with Heisenberg’s discovery of QM, inferred, sometimes by surmising them, from the physical features defining quantum phenomena and the principles or postulates, arising from these physical features. Technically, such principles would give rise to postulates. As Heisenberg said: “It should be distinctly understood that [the deduction of the fundamental equations of QM] cannot be a deduction in the mathematical sense of the word, since the equations to be obtained form themselves the postulates of the theory. Even though made highly plausible by the following considerations [leading to establishing QM], their ultimate justification lies in the agreement of their predictions with the experiment” [9] (p. 108). Thus, the formalism was a mathematical postulate made plausible in view of the physical postulates assumed and ultimately confirmed as correct by the experiment. So, as just noted, was Born’s rule. The mathematical scheme, initially proposed by Heisenberg [10] and fully developed into its full-fledged form, known as matrix mechanics, by M. Born and P. Jordan [11], was not using Hilbert spaces. Neither was E. Schrödinger’s (mathematically equivalent) form of it as wave mechanics. QM was recast into its Hilbert-space form a bit later by von Neumann [8]. Heisenberg’s postulates were as follow, building, especially in the case of (1), on those on Bohr 1913 atomic theory:

(1) The postulate of quantum discreteness, the QD postulate, according to which all observable quantum phenomena are discrete relative to each other (which is different from the atomic discreteness of quantum objects); 

(2) The postulate of the probabilistic or statistical nature of quantum predictions, the QP/QS postulate, maintained, in contrast to classical physics, even in considering individual quantum objects, and accompanied by the nonadditive character of quantum probability and rules, such as Born’s rule (a version of which was implicit in Heisenberg’s original paper), for predicting them; and

(3) The correspondence postulate, based in Bohr’s correspondence principle, which, as initially understood by Bohr, required that the predictions of quantum theory must coincide with those of classical mechanics in the classical limit, but was given by Heisenberg a mathematical form, postulating that the equations and variables of QM convert into those of classical mechanics in the classical limit.

Given that Heisenberg’s derivation of QM [10], which depended on (3), is not my concern here, I shall put (3) aside. On the other hand, (1) and (2), which have remained fundamental in quantum theory and is in Q-like theories as considered here, are central for my argument. Implicit in Heisenberg’s approach, especially in the first postulate was yet another postulate, central to quantum theory and to the present argument concerning Q-like models of human thinking. I shall designate it as the quantum individuality (QI) postulate, although quantum uniqueness conveys its nature even better. The QI postulate appeared explicitly, if not under this name, in Bohr’s “quantum postulate”, introduced, along with complementarity, in his so-called Como lecture of 1927 and grounding his first interpretation of quantum phenomena and QM. According to Bohr:

*Notwithstanding the difficulties … involved in the formulation of the quantum theory, it seems … that its essence may be expressed in the so-called quantum postulate, which attributes to any atomic process an essential discontinuity, or rather individuality, completely foreign to the classical theories and symbolized by Planck’s quantum of action [h].*

[12] (v. 1, p. 53)

This somewhat hesitant, yet necessary, “or rather” introduced one of the most essential features of quantum phenomena, the QI postulate, next to the QD postulate. Each quantum phenomena is strictly individual: it is unique and unrepeatable, just as is, as discussed below, every instance of human thinking. This essential discontinuity and individuality, both as equally necessary, will be, by the late 1930s, reinterpreted by Bohr in terms of his concept of phenomenon, defined strictly by what is observed in measuring instruments as a result of their interactions with quantum objects (e.g., [12], v. 2, p. 64).

Heisenberg, in considering electrons inside atoms, was not concerned with representing or even predicting, either (ideally) exactly or probabilistically, the motion of electrons. In effect, he abandoned the very concept of motion at the ultimate level of the physical reality considered. He was only concerned with predicting the probabilities of discrete transitions between the stationary (constant-energy) states of the electrons, which he no longer assumed to be represented, as they were in Bohr’s 1913 atomic theory, by the orbital motion of electrons around nuclei. Heisenberg added another important twist: “What I really like in this scheme is that one can really reduce all interactions between atoms and the external world … to transition probabilities” (Heisenberg, Letter to Kronig, 5 June 1925; cited in [13], v. 2, p. 242). QM was only predicting the effects of these interactions, observed in measuring instruments. This view was adopted by Bohr as the defining feature of his interpretation in all of its versions. I qualify because Bohr adjusted, sometimes significantly, his interpretation a few times. This requires one to always specify, as I will do here, to which version of his interpretation one refers. The designation “the Copenhagen interpretation” requires even more qualification as concerns whose interpretation it is specifically, say, that of Heisenberg, Pauli, Dirac, von Neumann and so forth what are its specific features, etc., which is why I shall avoid this designation altogether. 

J. Schwinger (who has his own interpretation of QM, which was close to, but still different from that of Bohr, especially Bohr’s ultimate interpretation, central to this article) instructively commented on why the formalism of QM was different from that of classical physics or relativity, in his unpublished lecture, cited at length in [14]. As is common (including in considering Q-models beyond QM), he stressed the fact that, if one measures two physical properties in one order, and then in the other, the outcome would in general be different, the fact linked to the noncommutativity of the corresponding variables, PQ−QP ≠0, a key new algebraic feature of QM, which was never used in physics previously. However, his aim was to explain the reasons for why this is the case, in effect in terms of QI and QD postulates. Before I consider his comments, I need to discuss two concepts implicit in them, the first is that of “classical causality”, and the second is that of quantum measurement, as this concept is understood in this article, following Bohr, to whom Schwinger is indebted in this respect as well. 

By classical causality I refer to the claim that the state, X, of a physical system is determined, in accordance with a law, at all future moments of time once it is determined at a given moment of time, state A and A is determined in accordance with the same law by any of the system’s previous states. This assumption also implies a concept of reality, which defines this law, thus making this concept of causality ontological. There are several reasons for my use of classical causality, rather than just causality, used more commonly in this context, including by Schwinger or Bohr. The main one is that it is possible to introduce alternative, including probabilistic, concepts of causality, applicable in QM where classical causality may not apply (e.g., [15], pp. 173–186; [16]). Some, beginning with P. S. Laplace, have used “determinism” instead or its avatars such as “deterministic causality”. I prefer to define “determinism” as an epistemological category referring to the possibility of predicting the outcomes of classically causal processes ideally exactly. In classical mechanics, when dealing with individual objects or small systems, both notions in effect coincide. On the other hand, classical statistical mechanics or chaos theory are classically causal but not deterministic in view of the complexity of the systems considered, which limit us to probabilistic or statistical predictions concerning their behavior. In the case of quantum phenomena, deterministic predictions are not possible even in considering the most elementary quantum phenomena, such as those associated with elementary particles. This is, as stated earlier, because the repetition of identically prepared quantum experiments in general leads to different outcomes, and in contrast with in classical physics, this difference cannot be diminished beyond the limit, defined by *h*, by improving the capacity of our measuring instruments. Hence, the probabilistic or statistical character of quantum predictions must be maintained by interpretations of QM or alternative theories of quantum phenomena that are classically causal. By contrast, RWR-type interpretations are not classically causal because the ultimate nature of reality responsible for quantum phenomena is assumed to be beyond a representation or even conception, while classical causality would imply at least a partial concept and indeed representation of this reality, in particular in terms of the classically causal behavior of quantum objects, defined by some laws at this level. The (mathematical) laws of QM or QFT, in RWR-type interpretations, are either predictive or apply to what happens in experiments, as manifested in quantum measurements, but not to what physically happens between experiments. If one aims at a purely mathematical representation of the ultimate nature of reality responsible for quantum phenomena, it is difficult to speak of such a representation as classically causal, because classical causality is, by definition, physical.

The concept of quantum measurement adopted in this article follows Bohr, eventually leading him to his concept of “phenomenon” (as applicable in quantum physics), as referring strictly to what has been observed or registered in measuring instruments. I shall discuss Bohr’s concept of phenomenon below. For the moment, in this understanding both concepts, quantum measurement and phenomenon, make the terms “observation” and “measurement”, as conventionally understood, inapplicable to the ultimate constitution of reality, as an RWR-type reality, responsible for quantum phenomena and thus to quantum objects, and indeed even to quantum phenomena. This term is a remnant of classical physics or still earlier history, from which classical physics inherited it, beginning with ancient Greek thinking and the rise of geometry there. In this view, a quantum measurement does not measure any property of this reality, which it would be assumed to possess before or even during the act of observation, as the concept of observation requires a different understanding as well. An act of observation in quantum physics establishes quantum phenomena by an interaction between the instrument and the quantum object. Then what is so observed as the data or information can be measured classically (in classical bits), just as one measures what is observed in classical physics, where, however, what is so measured could be associated with the object itself considered for all practical purposes. In on other words, as far as the observed data or information is concerned a quantum measurement is not a measurement of anything but a number or bit generator, a view that becomes crucial in quantum information theory and quantum computing, which enables computing without representing how a computation actually happens physically. In contrast with those of classical computing, the ultimate structure of quantum computing is not representable. We do not know how quantum computing ultimately compute, just as we do not know how an observation bring about what is observed in quantum phenomena. In the case of quantum phenomena, there is, thus, a difference between observations, which construct these phenomena, and measurements, which classically measure physical properties of these phenomena. While, however, keeping these qualifications in mind, I shall, for the sake of economy, just speak of measurement in quantum physics, rather than separating observation and measurement, which would be more precise.

I now return to Schwinger’s commentary on why the particular character of the quantum-mechanical formalism, including the use of noncommuting operator-observables, arises from the nature of quantum measurements, as just considered. According to Schwinger:

*Once we recognize that the act of measurement introduces in the [quantum] object of measurement changes which are not arbitrarily small, and which cannot be precisely controlled … then every time we make a measurement, we introduced a new physical situation and we can no longer be sure that the new physical situation corresponds to the same physical properties which we had obtained by an earlier measurement. In other words, if you measure two physical properties in one order, and then the other, which classically would absolutely make no difference, these in the microscopic realm are simply two different experiments …*

*So, therefore, the mathematical scheme can certainly not be the assignment, the association, or the representation of physical properties by numbers because numbers do not have this property of depending upon the order in which the measurements are carried out. … We must instead look for a new mathematical scheme in which the order of performance of physical operations is represented by an order of performance of mathematical operations.*

(Cited in [14], p. 361)

The last sentence is not entirely precise: mathematical operations, including multiplication (commutative or not), upon any quantities or symbols, are not physical measurements, which are, on the other hand, not mathematical, at least not inherently. This is an important point, of which Schwinger was undoubtedly aware. I shall return to this point presently. The passage contains echoes of Bohr’s writing. As Bohr said in his 1927 Como lecture: “It must not be forgotten … that in the classical theories any succeeding observation permits a prediction of future events with ever increasing accuracy, because it improves our knowledge of the initial state of the system. According to the quantum theory, just the impossibility of neglecting the interaction with the agency of measurement means that every observation introduces a new uncontrollable element” [12] (v. 1, p. 68). Classically, one can continue to perform measurements of both the position and the momentum of an object at any point along its continuous and classically causal trajectory. This is not possible in quantum measurements, even if one assumes, as one does, for example, in Bohmian mechanics, that such a trajectory and classical causality are possible. The role of “the finite [hence not arbitrarily small] and uncontrollable interaction” between the object and the instrument was emphasized by Bohr as well [17] (pp. 697, 700).

The main difficulty of quantum measurement, manifested in Schwinger’s argument, is that, given this situation, any mathematical scheme defining the formalism of quantum theory does not appear to be representationally connected, in the way it would be in classical physics or relativity, to “measurement algebra”, as Schwinger came to call it [18,19]. Consider Schwinger’s statement: “the order of performance of physical operations is represented by an order of performance of mathematical operations”. If one follows Bohr, as Schwinger appears to do in his lecture, these physical operations are measurements, with their outcomes, manifested in measuring instruments. These measurements are predicted by means of these mathematical operations, but are not represented by them, in the way they would be in classical physics. As just explained, a quantum measurement is not a measurement of some preexisting quantity of the quantum object considered but an establishment of a new quantum phenomenon, an entirely “new physical situation”, as Schwinger says. Accordingly, the term “represented” (perhaps loosely used by Schwinger) is not accurate insofar as there is no homomorphic, let alone isomorphic, mapping from the algebra of QM, “the new mathematical scheme”, to this measurement algebra, to the degree it is an algebra, a point I shall address presently.

The assessment just given reflects a very different understanding of the structure of quantum measurement from that of assuming that the noncommutative nature of the operator variables involved, PQ−QP ≠0, represents the order of performance of physical operations. This understanding arises from Schwinger’s key premises here: “Once we recognize that the act of measurement introduces in the [quantum] object of measurement changes which are not arbitrarily small, and which cannot be precisely controlled … then every time we make a measurement, we introduced a new physical situation and we can no longer be sure that the new physical situation corresponds to the same physical properties which we had obtained by an earlier measurement”. By the QI postulate, each quantum measurement is a unique and unrepeatable event defining a new physical situation. As such, each new measurement, *M*_2_ at a late moment in time *t*_2,_ even of the same variable, unavoidably requires, in Schrödinger’s terms, a new expectation-catalog, enabled by QM cum Born’s rule, and the data obtained in the previous measurement, *M*_1_, at an earlier time *t*_1_ [20] (p. 154). This new measurement, as a unique event, even if we measure the same variable, say, the position, makes the previous expectation-catalog, defined by *M*_1,_ meaningless as concerns possible predictions after *M*_2_ is made. If the same measurement, *M*_1,_ is repeated instantly the outcome may be and commonly is assumed to be the same, as a permissible idealization. 

Let us now consider the case of the measurement of two complementary variables, say, the position and the momentum, associated with a quantum object, the case in which the noncommutative algebra of the formalism comes into play. It may be useful to explain the concept of complementarity first, in order properly to appreciate that the complementary variables of QM (although still conjugate variables mathematically) are not the same as the conjugate variables of classical mechanics. Defined arguably most generally, complementarity is characterized by:

(a). a mutual exclusivity of certain phenomena, entities or conceptions, and yet;
(b). the possibility of considering each one of them separately at any given point;
(c). the necessity of considering all of them at different moments of time for a comprehensive account of the totality of phenomena that one must consider in quantum physics.

The concept was never given by Bohr a single definition of this type. However, this definition may be surmised from several of Bohr’s statements, such as: “Evidence obtained under different experimental conditions cannot be comprehended within a single picture but must be regarded as complementary in the sense that only the totality of the phenomena [some of which are mutually exclusive] exhaust the possible information about the objects” [12] (v. 2, p. 40; emphasis added). In classical mechanics, we can comprehend all the information about each object within a single picture because the interference of measurement can be neglected: this allows us to (ideally) identify the phenomenon considered with the object under investigation and to establish the quantities defining this information, such as the position and the momentum of each object, in the same experiment. In quantum physics, this interference cannot be neglected and defines any quantum phenomenon. This interference leads to different experimental conditions for each measurement on a quantum object and their complementarity, in correspondence with the uncertainty relations. The situation implies two incompatible pictures of what is observed, as phenomena, in measuring instruments. Hence, the possible information about a quantum object, the information to be found in measuring instruments, could only be exhausted by the mutually incompatible evidence obtainable under different experimental conditions. In fact, possible information associated with a quantum system is always incomparably larger than that which can (always as classical information) be actually obtained, extracted, by a measurement. On the other hand, once made, either measurement, say, that of the position, will provide the complete actual information about the object, as complete as possible, at this moment in time. One could never obtain the complementary information, provided by the momentum measurement, about this object at this moment in time, because in order to do so one would need simultaneously to perform a complementary experiment on it, which is not possible. At the same time, it is crucial, that, by (b) one can decide to perform either one or the other measurement at any point. Thus, complementarity is underlain by both QI and QD postulates, which grounded complementarity all along, beginning with Bohr’s quantum postulate of 1927 considered above.

Now, sequential measurements of complementary variables, measurements observed in the measuring instruments used, are mutually exclusive as concerns the possibility of performing both simultaneously, by virtue of the uncertainty relations, which are experimentally established laws independent of any theory, but reflected in the corresponding complementary quantum phenomena. If the first measurement, *M*_1_, at time *t*_1_, is that of a coordinate (observed in a measuring instrument), one can make predictions, by using QM cum Born’s rule, concerning the probability or statistics of a future position measurement at any future moment in time, *t*_n_, but can make no predictions whatsoever concerning future momentum measurements at any future moment in time. However, if we then make the momentum measurement (in the same direction), say, *M*_2_, at time *t*_2_, our earlier predictions concerning the position measurement at this point become meaningless as one can no longer verify it, while this new measurement, now, precludes us from making any predictions concerning future position measurements. One can only make predictions concerning future momentum measurements, which will find the value of the momentum with a given probability within a certain range.

The noncommutativity of the corresponding operator variables, *Q* and *P*, is just part of the mathematics that enable either predictions, rather than representing this situation of measurement, or this “algebra of measurement”, to the degree one could speak of “algebra” here, rather than of the structure of measurement, defined by this situation or by quantum measurement in general. It is true that, if in the experiment with the initial preparation of measuring instruments at time *t*_01_, one makes first the position measurement, *M*_1*Q*,_ at time *t*_11_ and then the momentum measurement, *M*_2P_ at time *t*_21_, and then, with the same initial preparation of measuring instruments at time *t*_02_ (which preparation is possible because we can control the instruments classically) reverse the order of the quantities one measures, by first measuring the momentum, *M*_1*P*,_ at time *t*_12_ and then the position at time *t*_22_, *M*_2*Q*_, the outcome will be different. The double indexing of time is necessary because each set of measurements happens at different set of time intervals and in fact require a different quantum object. This situation is the physical meaning of Schwinger’s appeal to two different situations of measurements, the meaning defined by the fact that each situation consists of two separate measurements. One is dealing with two pairs of different physical entities, each unique and unrepeatable, by the QI postulate, (*M*_1*Q*_, *M*_2*P*_) and (*M*_1*P*_, *M*_2*Q*_) observed in measuring instruments, and not with performing a reverse (“algebraic”) operation with different outcomes on the same two entities, *M*_1_ and *M*_2_, as one would mathematically in noncommutative algebra, with *M*_1_ *M*_2_ ≠ *M*_2_ *M*_1_. Of course, PQ−QP ≠0 for all complementary variables considered, but this mathematical fact only pertains the formalism of QM, enabling correct probabilities and statistics of predictions, by using these Ps and Qs, but not to measurements. These variables are, at least in the present or Bohr’s (or Schwinger’s) view, otherwise independent of the physical quantities measured, while the latter are, conversely, independent of any specific theory, as are, again, the uncertainty relations. 

An analogous, although not identical, situation emerges in dealing with our interference, “measurement”, from outside or even from within our mind, into thinking processes. One example of this interference is when the order of our decisions, say, in response to two questions asked in the reverse order, affects the outcomes. Such situations were among the motivations for using Q-like models in psychology, as in the paradigmatic case asking two questions, “Do you generally think President Clinton is honest and trustworthy?” and “Do you generally think Vice President Gore is honest and trustworthy?” in different order, which often, but (as against quantum physics) not always, yield different answer (e.g., [21,22,23]). Most crucial, however, is a more general fact under discussion, defined by the QI postulate—that (generalizing Schwinger’s formulation), “once we recognize that the act of interference introduces in the behavior of the object under investigation changes which are not arbitrarily small, and thus essentially discrete, and which cannot be precisely controlled … then every time we make an interference, we introduce a new experimental situation, a new phenomenon, and we can no longer be sure that the new situation corresponds to the same properties which we had obtained by an earlier interference”. This view and thus the QI postulate could, I argue, be strictly maintained in considering human thinking and decision making. Thus, both areas of investigation share the QI postulate, and other principles and postulates, such as QD and QP/QS postulate, or the irreducible role of observational agency in the constitution of the phenomena considered.

At the same time, there are differences in the ways these principles and postulates work in considering quantum phenomena and quantum-like phenomena in psychological domains. To indicate one of them, arguably the most decisive one, in contrast with human subjects, physical objects do not have consciousness (C) or the unconscious (UC), or C-UC systems of thinking. Some do suggest, for example, in physicalist Q-like approaches to the hard problem, that quantum objects do possess something akin to consciousness and even make decisions. Such views will be put aside here. Before, however, I turn to Q-like aspects of human thinking, I shall outline an interpretation of quantum phenomena and QM (or QFT), which responds to the features of quantum phenomena and these theories here discussed. As stated from the outset, this interpretation is nonrealist and is defined specifically (there is no single form of nonrealism any more than realism) by the concept of reality without realism, RWR, which still allows for a spectrum of possible interpretations. The same interpretation will then be considered, in correlative terms of ideality without idealism, IWI, in the case of psychological phenomena and Q-like theories dealing with them.

## 3. Reality, Realism and Reality without Realism in Quantum Theory

Quantum phenomena were initially defined by the fact that in considering them, Planck’s constant, *h*, must be taken into account. While, however, measuring any quantum phenomenon known thus far involves *h*, its role may not be sufficient to fully distinguish quantum phenomena from classical ones, and their specificity as quantum appears to be defined by a broader set of features, some of which are not expressly linked to *h*. Some of these features are exhibited by classical phenomena or found in toy theories different from QM. Accordingly, one now has a more complex sense of quantum phenomena. Thus, the following, sometimes correlative, features of quantum phenomena appear all to be necessary to define them vs. classical phenomena—(1) the role of *h*, (2) the irreducible role of measuring instruments in defining quantum phenomena, (3) discreteness, (4) individuality, (5) complementarity, (6) entanglement, (7) quantum nonlocality and (8) the irreducibly probabilistic or statistical nature of quantum predictions. If one adopts an RWR-type interpretation, it is tempting to assume, following Bohr, that, if there is any single feature distinguishing classical and quantum phenomena, it is the irreducible interference of measuring instruments in defining quantum phenomena. This feature is also central for my argument concerning human thinking and using Q-like theories in considering it because one interferes with mental processes, both from the inside (the unconscious may interfere with consciousness) and the outside. However, one might prefer to err on the side of caution. The degree to which all of these features could be transferred to quantum-like phenomena is a separate matter, but some of them, such as (2) (3), (4), (5) and (8) can, which leads to the use of Q-like theories there. There is no *h*, irreducible in all quantum phenomena, or comparable numerical constants, in, it appears, Q-like phenomena considered thus far. 

By quantum theory I refer to a set of conceptual schemes accounting for quantum phenomena. The history of any theory is accompanied by the history of its interpretations, most generally, defined by concepts added to a theory, such as those that establish how the theory refers to the phenomena it considers. I define a mathematical model in physics as a mathematical structure or a set of mathematical structures that enable such relationships. As that of a theory, the concept of a mathematical model has a long history and diverse definitions. It is not my aim to discuss the subject as such or engage with literature addressing it, which would be difficult to do and is not necessary for my aims in these articles [15] (pp. 5–6) [24]. The present concept of a mathematical model, while open, is sufficient to accommodate those models that I consider here. The history of QM has been shaped by a seemingly uncontainable proliferation of interpretations. It is not possible to survey these interpretations here. Even each rubric, on by now a long list (e.g., the Copenhagen, the many-worlds, consistent-histories, modal, relational and so forth), contains different versions, sometimes quite a few of them. The situation is only barely more manageable in QFT.

I shall now outline the concept of reality without realism (RWR) and the corresponding view of the nature of reality, the RWR view, which grounds the present and related interpretations, such as that of Bohr in its ultimate version (Bohr, as I said, changed his views a few times) of quantum phenomena and QM, as well the present interpretation of psychological phenomena. Bohr made some suggestions in this direction as well, but tentative in nature, considered in [25] (pp.156–166). This concept was introduced by this author previously [15,26,27]. It is grounded in more general concepts of reality and existence, assumed to be primitive concepts and not given analytical definitions. By “reality” I refer to that which is assumed to exist, without making any claims concerning the character of this existence. The absence of such claims, which define realism, allows one to place this character beyond representation or even conception. I understand existence as a capacity to have effects on the world with which we interact. The very assumption that something is real, including of the RWR-type, is made on the basis of such effects. 

I outline first realist thinking, manifested in the corresponding theories, which are commonly representational in character. Such theories aim to represent the reality they consider, usually by mathematized physical models based on suitably idealizing this reality. It is possible to aim, including in quantum theory, for a strictly mathematical representation of this reality apart from any physical concepts, the situation that, as will be seen, is found in Q-like theories. It is also possible to assume an independent structure of the reality considered, while admitting that it is either (A) not possible to adequately represent this structure by means of a physical theory or (B) even to form a rigorously specified concept of it, either at a given moment in history or even ever, while still assuming that this reality has a structure. Under (A), a theory that is merely predictive could be accepted for lack of a realist alternative, but usually with the hope that a future theory will do better by being a properly representational theory. Einstein adopted this attitude toward QM or, by implication, QFT, which he expected to be eventually replaced by a realist theory. What, then, grounds realism most fundamentally is the assumption that the ultimate constitution of reality possesses properties and the relationships between them, or, as in (ontic) structural realism [28], just a structure that may either be ideally represented and hence, known by a theory or be unrepresentable or unknown or even unknowable, but still conceivable, usually with a hope that it will be eventually so represented. 

Thus, classical mechanics (used in dealing with individual objects and small systems, apart from chaotic ones), classical statistical mechanics (used in dealing, statistically, with large classical systems), chaos theory (used in dealing with classical systems that exhibit a highly nonlinear behavior) or relativity, special and general, are realist theories. While classical statistical mechanics does not represent the overall behavior of the systems considered because their great mechanical complexity prevents such a representation, it assumes that the individual constituents of these systems are represented by classical mechanics. In chaos theory, which, too, deals with systems consisting of large numbers of atoms, one assumes a mathematical representation of the behavior of these systems directly. All these theories are based in the assumption that we can observe the phenomena considered without disturbing them. As a result, we can identify these phenomena with the corresponding physical objects and their independent behavior and (ideally) represent and predict this behavior by using this representation, keeping in mind qualifications necessary for classical statistical physics or chaos theory.

RWR-type thinking may be seen as grounded in the fact that ascertaining observable effects of physical reality entails a representation of these effects but not necessarily a representation or even a conception of how these effects come about. Such a representation may not be possible and it is not in the RWR view. Conversely, however, while one cannot represent (in which case I shall speak of the weak RWR view) or even think, conceive of (in which case I shall speak of the strong RWR view), an RWR-type reality, one can think, know and represent its effects. As discussed in Section 4, the concept of reality without realism can apply in mental domains, for example, in psychology or mathematics. An RWR-type theory or interpretation might and even must assume different levels of idealizations of reality, some allowing for a representation or conception and others not. As noted, Bohr was the first to ground his interpretation (in all of its versions) in the irreducible role of measuring instruments in the constitution of quantum phenomena, observed classically and, in its ultimate version, in the RWR-type concept of reality as applied to the ultimate constitution of the reality responsible for quantum phenomena.

This constitution is commonly, including in Bohr’s interpretation, associated with quantum objects. The interpretation (of the strong RWR-type) adopted here, however, takes a more stratified view of the situation, which is, as will be seen, also more fitting to considering human thinking and decision making. This situation is as follows. The ultimate RWR-type reality responsible for quantum phenomena is an idealization assumed to exist independently of our interactions with it, and thus independently of observation or measurement. On the other hand, the reality idealized as quantum objects is, while still of the RWR-type, assumed to exist only at the time measurement, defined, as a creation of quantum phenomena, by the interactions between this ultimate RWR-type reality and measuring instruments. In this interpretation, there are no quantum objects existing independently in nature apart from our interaction with it, which redefines the concept of quantum object, including as against Bohr’s view. Hence, one cannot speak of the behavior of quantum objects as independent of measurement, which makes quantum objects, on this point following Bohr, physically indissociable from quantum phenomena (e.g., [12], v. 2, pp. 61, 72). 

The behavior of the observable parts of measuring instruments, defining quantum phenomena, is, thus, idealized as representable, ultimately by means of classical physics. Measuring instruments, however, also have quantum strata through which they interact with quantum objects. To anticipate my argument below, our representational thinking, or more accurately representations created by our thinking, conscious or unconscious, appears to be essentially classical-like, that is, the type of general phenomenal representation shared with, but conceptually and mathematically refined, by classical physics. Eventually, Bohr adopted the term “phenomenon” to refer strictly to what is observed under specified experimental setups, in measuring instruments, as effects of their interaction with quantum objects, or more precisely what has been so observed. Defined by “the observations [already] obtained”, phenomena refer to events that have already occurred and not to future events that one can predict on the basis of previous events [12] (v. 2, p. 64).

The reason for assuming the present, more stratified, idealization, rather than seeing the situation only in terms of quantum phenomena and quantum objects (a more Kantian view) is as follows. While, in contrast to classical physics or relativity, in quantum physics, in each experimental arrangement one must, in Bohr’s words, discriminate “between those parts of the physical system considered which are to be treated as measuring instruments and those which constitute the objects under investigation”, the difference between them is, in general, not uniquely defined [17] (p. 701). This is expressed as the arbitrariness of the “cut”, discussed below. Accordingly, it is how we set up an experiment that defines what is the quantum object in this experiment and thus brings this object into existence. This view can, and here will, be transferred to our understanding of human experience as unique and unrepeatable at any given point of time in any observation of from the inside or (this is always indirect) from the outside, just as in any quantum experiment. In this case, moreover, there are no entities analogous to quantum objects, such as electrons, photons or (as they may also be composite) protons, whose type-identity could be maintained in different experiments. There are only phenomenal representations observed, from within or without, as always ultimately different. The observed states of quantum objects are different, too. However, while mental representations can have parts shared between different subjects, overall, they are ultimately different for each subject and this difference can come into play in an experiment. In quantum physics, a measured physical state of, say, an electron is always that of an electron. In high-energy (QFT) quantum regimes, in contrast with low-energy (QM) regimes, one can in the same experiment register different particles, which, as discussed later, make it a better Q-like model for human thinking. Still, however, one deals within a determinable finite array of objects, while thinking present us with an uncontainable manifold of possibilities in each observation, which may not be captured by QFT-like mathematical models. 

There is still the question of whether our inability, in assuming a strong RWR-type interpretation, to conceive of the ultimate nature of reality responsible for quantum phenomena (and in this present view, also quantum objects) only (A) characterizes the situation in quantum physics as things stand now, while allowing that quantum phenomena or whatever may replace them as fundamental phenomena will no longer make this assumption and thus RWR-type interpretations viable, reverting to a realist view; or (B) reflects the possibility that this reality will never become available to thought. Obviously, one cannot claim more than a possibility here. Logically, once (A) is the case, then (B) is possible, but is not certain. There does not appear to be any experimental data compelling one to prefer either. They are, however, different philosophically in demarcating how far our mind can, in principle, reach in understanding nature, or possibly the mind, which is why I mention both. While Bohr, at least assumes (A) and while some of his statements suggest that he entertained (B), he never expressly stated so, which leaves whether he assumed (B) or only (A) to interpretation.

One of the reasons for entertaining (B) is that our biological and neurological constitution, and, thus, our thinking and language, enabled by this constitution, have evolutionarily developed and could only have developed in our interaction with objects in the world consisting of billions of atoms and thus became essentially classical-like as regards any representation it can form. More accurately, again, these representations are something that classical physics mathematically refines, a point emphasized by both Bohr and Heisenberg (e.g., [9] (pp. 11, 64–65) and [12] (pp. 69–69)) and supported by recent neurological research (e.g., [29]). It follows that there is no special reason to assume that we should be able to describe how nature ultimately works at its very small (or very large) scales, thus, possibly putting ultimate limits on how far our thinking could reach in our understanding of nature. 

The circumstances outlined here imply a different reason for the recourse to probability in quantum physics in RWR-type interpretations. According to Bohr:

*[I]t is most important to realize that the recourse to probability laws under such circumstances is essentially different in aim from the familiar application of statistical considerations as practical means of accounting for the properties of mechanical systems of great structural complexity [in classical physics]. In fact, in quantum physics we are presented not with intricacies of this kind, but with the inability of the classical frame of concepts to comprise the peculiar feature of indivisibility, or “individuality”, characterizing the elementary processes.*

[12] (v. 2, p. 34)

Rather than representing a definitive state of affairs, this statement should be seen as expressing the strong RWR-type interpretation adopted by Bohr at this point, in 1949. Some interpretations of QM, such as those by Dirac [30] and von Neumann [8], or alternative theories, such as Bohmian mechanics (in a very different way), assume classically causal views of the behavior of quantum objects, with probability or statistics brought in by measurement. Both individuality, here expressed in the QI postulate, and “indivisibility” reflect Bohr’s concept of phenomena. “The classical frame of concepts” may appear to refer to the concepts of classical physics, and it does include these concepts. By this time (in 1949), however, Bohr adopts the strong RWR view, which places the ultimate nature of reality responsible for quantum phenomena beyond conception. This gives the phrase “the classical frame of concepts” a broader meaning, indicated above: All representational concepts that we can form are classical-like, insofar as the physical concepts of classical physics are (mathematized) refinements of our phenomenal intuition, which, along with our language, is a product of our neurological machinery, developed in the course of evolution. This refinement may no longer be available for representing the ultimate nature of reality responsible for quantum phenomena or the ultimate constitution of nature in general, or, as I shall suggest, in closing this article, perhaps of thinking as well, even if considered strictly in mental terms.

I close this section by returning to the structure of quantum measurement adopted here, in part following Bohr. According to Bohr:

*This necessity of discriminating in each experimental arrangement between those parts of the physical system considered which are to be treated as measuring instruments and those which constitute the objects under investigation may indeed be said to form a principal distinction between classical and quantum-mechanical description of physical phenomena. It is true that the place within each measuring procedure where this discrimination is made is in both cases largely a matter of convenience. While, however, in classical physics the distinction between object and measuring agencies does not entail any difference in the character of the description of the phenomena concerned, its fundamental importance in quantum theory … has its root in the indispensable use of classical concepts in the interpretation of all proper measurements, even though the classical theories do not suffice in accounting for the new types of regularities with which we are concerned in atomic physics. In accordance with this situation there can be no question of any unambiguous interpretation of the symbols of quantum mechanics other than that embodied in the well-known rules which allow us to predict the results to be obtained by a given experimental arrangement described in a totally classical way.*

[17] (p. 701)

Before I consider this situation itself, I want to address two common misunderstandings of this and related statements by Bohr. First, Bohr’s statement may suggest that, while observable parts of measuring instruments are described by means of classical physics, the independent behavior of quantum objects is described or represented by means of the quantum-mechanical formalism. This type of view has been adopted by some, for example, as noted earlier, Dirac [30] and von Neumann [8], whose unitary ontology (“the unitarity dogma”) was convincingly critiqued by M. G. D’Ariano [31]. It was not, however, Bohr’s view, at least after he revised his argument offered in the article, known as the Como lecture, that introduced his first full-fledged interpretation of quantum phenomena and QM [12] (v. 1, pp. 52–91). The Como lecture entertained (still ambivalently) this type of view and which had influenced others, including Dirac and von Neumann. In the above elaboration, Bohr does say that the observable parts of measuring instruments are described by means of classical physics and that classical theories cannot suffice to account for quantum phenomena. However, he does not say that the independent behavior of quantum objects is represented by quantum-mechanical formalism. His statement only implies that quantum objects cannot be treated classically, which is a very different claim. The “symbols” of quantum-mechanical formalism are assumed, as they always are by Bohr, only to have a probabilistically or statistically predictive role.

Secondly, Bohr’s insistence on the indispensability of classical physical concepts in considering measuring instruments is often misunderstood as well, in particular by disregarding that measuring instruments contain both classical and quantum strata. Even though what is observed as phenomena in quantum experiments is beyond the capacity of classical physics to account for them, the classical description can and, in order for us to be able to give communicable accounts of what happens in quantum experiments, must apply to the observable parts of measuring instruments. The instruments, however, also have the quantum stratum, through which they interact with quantum objects, which interaction would not be possible otherwise. This interaction is quantum and cannot be observed or, in RWR-type interpretations, represented or even conceived of.

One could attempt to formalize this situation, as, for example, it was in by M. Ozawa [32,33]. One considers a compound quantum system, QO + QI, consisting of the quantum object under investigation, QO and the quantum part, QI, of the instrument I (QO + QI), which is isolated during the (short) time interval when the quantum interaction in question takes place. The rest of the instrument, I, performs the measurement, a pointer measurement, on QI, after the interaction has taken place. In realist schemes, such as that of M. Ozawa, the evolution of the QO + QI, the unitary evolution operator, $U\left(t\right)=e^{-i\Delta tH}$, where *H = H_QO_ + H_QI_ + H_QOQI_* is the Hamiltonian representing the internal behavior of the subsystems involved and *H_QOQI_* the interaction between them. As will be seen, Khrennikov uses this scheme in considering the interaction between the unconscious and consciousness, assumed to measure the unconscious [34], which is the main reasons why I refer to Ozawa’s works, as opposed to similar earlier formalizations of quantum measurements, such as by G. Ludwig [35] and P. Mittelstaedt [36]. In Bohr’s and the present view, as strong RWR views, no element of the formalism ever represents the ultimate reality, including that of the interaction between QO and QI, responsible for the effects observed. Any such element is only part of the mathematical machinery of QM that, with the help of Born’s rule, predicts such effects. 

The situation under discussion is sometimes referred to as the arbitrariness of the “cut” or, because the term [Schnitt] was favored by Heisenberg and von Neumann, the “Heisenberg-von-Neumann cut”. As Bohr noted, however, while “it is true that the place within each measuring procedure where this discrimination [between the object and the measuring instrument] is made is … largely a matter of convenience”, it is true only largely, but not completely. This is because “in each experimental arrangement and measuring procedure we have only a free choice of this place within a region where the quantum-mechanical description of the process concerned is effectively equivalent with the classical description” [17] (p. 701). Thus, the ultimate constitution of the physical reality responsible for quantum phenomena, observed in measuring instruments, is never on the measurement side of the cut. Neither are quantum strata of the instruments through which the latter interact with this reality.

As stated earlier, in the present view, even if not that of Bohr, a quantum object, while still an RWR-type idealization, is different from that of the ultimate, RWR-type, reality, that is responsible for both quantum objects and quantum phenomena. While a measuring instrument, which is, in its observable part, a classical object or, at the other pole, the ultimate RWR-type reality considered, are assumed to exist independently, a quantum object can, in view of these considerations, only be rigorously ascribed existence and be defined by a measurement and its setup, including the cut, and thus by our observation of the outcome of this measurement. Accordingly, as noted, in this view there is no independent behavior of quantum objects: there is only the interaction between the ultimate (RWR-type) nature of reality and measuring instruments, which interaction allows one to define quantum objects. What is a quantum object in a given experiment can be different in each case, including possibly something that, if considered by itself, could be viewed as classical, as in the case of Carbon 60 fullerene molecules, which were observed as both classical and quantum objects [37]. The quantum nature of any quantum object is still defined by its microscopic constitution. The ultimate reality responsible for this situation is, again, assumed to have an independent existence and is, always, on the other side of the cut. 

The following question might, then, be asked. If a quantum object is only defined by an experiment or measurement, rather than as something that exists independently, could one still speak of the same quantum object, say, the same electron, in two or more successive measurements? Consider (speaking in more conventional terms) two position measurements, the first defined by a slit in a diaphragm through which an electron, emitted from some source, may be assumed to be registered to pass by some counter, and the second defined by a collision between it and a silver bromide screen at some distance from the diaphragm. These two measurements may be seen as two parts of a single experiment, the first sometimes referred to as a preparation. According to the view here outlined, each of these two measurements defines an electron, with the same mass and charge, in two different positions at two different moments in time. The case can be given a strictly RWR interpretation, insofar as all these properties (mass, charge and position) are, physically, those of measuring devices, assumed to be impacted by quantum objects, rather than of these objects themselves, placed beyond representation or conception. For the moment, the question is: Do these two measurements register the same electron? Rigorously speaking, if the idealization of quantum objects is only applicable at the time of measurement, then a prediction based on a given measurement and the new measurement based on this prediction could only concern a new quantum object, and not an object that we measured earlier in making a prediction. Accordingly, rigorously, one deals with two different quantum objects, two different electrons, for example. To consider them as the same electron is, however, a permissible idealization in low-energy (QM), or low-energy QFT, regimes, an idealization ultimately statistical in nature, because a detection of the electron in the second measurement is not guaranteed, although the probability that it will not occur is low. On the other hand, speaking of the same electron in any two successive measurements in high-energy (QFT) regimes is meaningless, because two such measurements can register quantum objects of different type, say, an electron in the first and a photon in the second. This circumstance further justifies the present concept of a quantum object and the tripartite idealization scheme just outlined.

In considering human thinking, an observation, from within (by the consciousness of a given human subject) or without (by an outside agent), could register representational entities, such as statements, that could be treated as identical for certain purposes. They will, however, always be parts of unique and unrepeatable states of thinking, which can give them different meanings, as related to different arrays of qualia (qualitative phenomenal properties). The “cut” between conscious and unconscious thinking and resulting representations would acquire the corresponding complexities as well. On the other hand, such features as the irreducible role of observation in defining the phenomena considered, the QI postulate, the QD postulate and the QP/QS postulate, could still be maintained in theorizing, in a Q-like way, human thinking.

## 4. “Most Tantalizing State of Affairs”: Why Is Human Thinking Quantum-Like?

I am working out a quantum theory about it for it is really most tantalizing state of affairs.

—James Joyce, *Finnegans Wake* [38] (p. 149)

The answer to the question posed by this section’s subtle, or at least the reason for asking it, is in the circumstance, supported by research in several fields, that of quantum phenomena in physics and at least some phenomena associated with human thinking (not all phenomena are quantum in physics either) and theories dealing with these phenomena, share certain fundamental principles, postulates and concepts considered in this article in the case of quantum phenomena. The most essential among them for the present argument are the following: the irreducible role of observation in defining the phenomena considered, the QI postulate (the irreducible individuality or uniqueness of quantum phenomena), the QD postulate (the irreducible discreteness of quantum phenomena) and the QP/QS postulate (the irreducibly probabilistic or statistical character of our predictions concerning quantum phenomena). While several other features of quantum physics discussed here, such as and in particular complementarity, are just as important, they are consequences of, or responses to, these more fundamental postulates. The pertinence of analogous principles, postulates and concepts also makes it possible to apply them and, as a result, interpretively, the RWR view to our understanding of human thinking and decision making. At the same time, the functioning and even certain aspects of these principles, postulates and concepts change when our objects of enquiry are human subjects, rather than physical objects. It particular, as noted earlier, physical objects, classical or quantum, do not have consciousness (C) or the unconscious (UC), or C-UC systems, defining human thinking, and on the other hand, in dealing with human subjects as objects of inquiry, we do not have identical objects as we do in quantum theory, elementary, such as photons, electrons or neutrino, or composite, such as protons or neutrons, hadrons or chemical elements, beginning with hydrogen. 

I want to, first, briefly return the role of human decision in dealing with quantum phenomena by means of QM, and then compare it with dealing with phenomena associated with human thinking. I put QFT aside for the moment, assuming that the situation there is not different in this regard, as opposed to the difference in possible outcomes of experiments, more complex in QFT, which may make QFT-like theories better models of human thinking, a point considered below. As discussed earlier, in quantum physics, our decisions concerning which experiments we perform, always, in all possible cases, essentially affect, by interfering with reality, what may or, for example, by complementarity, may not happen with one probability or another. As a result, our decisions affect the course of reality, in contrast to classical physics or relativity, where our experiments, generally, follow what would happen in any event. Our experiments may affect the course of reality there in some cases, but in quantum physics they do so in all cases. There may be a question how much of a “free choice” we have in such decisions and within what limits. The so-called “superdeterminism” denies that we ever do [39]. I do not subscribe to this view. On the other hand, there are factors (interior and exterior) that limit and sometimes preclude our freedom of choice, which make the category of decision preferable to that of (free) choice. A philosophically more complex and more charged question of free will be put aside here as well. Doing so may be seen as a limitation, but this decision is due to my main argument, which concerns the role of the principles, postulates and concepts listed above in Q-like approaches to human thinking. I want to, however, cite Bohr, who, in describing complementarity, specifically that of the position and the momentum measurements, speaks of “our freedom of handling the measuring instruments” (although, I think, that he would have accepted the qualifications just given concerning this freedom):

*In the phenomena concerned we are not dealing with an incomplete description characterized by the arbitrary picking out of different elements of physical reality at the cost of [sacrificing] such elements, but with a rational discrimination between essentially different experimental arrangements and procedures which are suited either for an unambiguous use of the idea of space location, or for a legitimate application of the conservation theorem of momentum. Any remaining appearance of arbitrariness concerns merely our freedom of handling the measuring instruments, characteristic of the very idea of experiment.*

[17] (p. 699)

One, then, decides, say, at time *t*_1_, which experiment to perform, either the one concerning the value of the position or that concerning the value of the momentum. This decision then enables one, by using QM, to establish the probability of finding, within a certain range, either the position or the momentum of the object at any subsequent moment in time, *t_2_*. Either measurement in principle precludes predicting such a probability for the complementary variable. If one performs two such measurements in sequence, the outcomes will be different depending on the order in which they are performed, because each creates a different, unique measurement situation, by the QI postulate. As discussed in Section 2, actually performing these two sequences of measurement requires two different experimental situations and two different quantum objects. This fact, underlain by the irreducible role of measuring instruments and the QI postulate, is itself a reflection of complementarity insofar as these two initial measurements and these two alternative sequences are mutually exclusive. In contrast with in classical physics or relativity (where the interference of measurement can be neglected and, as a result, the QI and QD postulates do not apply) in quantum physics we always investigate the compound system consisting of the quantum object and the instrument, which has both a classical observable part and a quantum part enabling its interaction with the object. As explained, this irreducible role of measurement in the constitution of quantum phenomena makes the QI postulate and, correlatively, the QD postulate unavoidable even in realist interpretations. 

Now, while these postulates still apply, the case is different in dealing with human thinking because the objects under investigation as well as the measuring instruments are human subjects. Consider, again, the case, paradigmatic in quantum-like approaches to human thinking, of asking two questions, “Do you generally think President Clinton is honest and trustworthy?” (which asked by itself tends to elicit the negative answer) and “Do you generally think Vice President Gore is honest and trustworthy?” (commonly answered positively) (e.g., [21,22]). First, just as one would in QM, an agent conducting the experiment or survey makes a decision, an A-decision (A for “agent”), in which order to ask these questions, which is not so different from quantum experiments concerning one or the other order of measuring two complementary variables. The main difference from quantum physics emerges when one considers the C-UC system of each human subject asked, and the experiment in question at the moment, of course, concerns the decisions (defining answers) by these subjects. Each human subject considered, defined by a C-US system, also makes a decision in answering each question, an S-decision, with S standing for “subject”. This decision is conscious, although the unconscious of the subject may affect or even define it, thus bringing in the C-UC system. This difference from quantum physics remains in place, even though in quantum physics, one, in each case, also investigates the system consisting of the object and the instrument, because neither is a human subject. There have been arguments that viewed quantum objects as differently “responding” to different experimental setups or, as noted earlier, even possessing something akin to consciousness. Putting aside the latter view altogether, I am hesitant to ascribe such human attributes as “responding” to nature, but instead see the situation in terms of different types of interactions between quantum objects and instrumental setups.

Even though in psychological experiments or poling practices, A-decisions are generally determined in advance, one still deals with the interplay of two types of decisions, A and S, determined by C-UC systems, and this fact comes into play in considering human thinking and decision making in general. A human subject can have an opinion concerning both Clinton and Gore simultaneously and one can establish both simultaneously, by asking “Do you trust both Clinton and Gore?” and receiving yes and no answers, if one insists on a single answer, and one can also receive split answers otherwise. One does not do so in the standard setup of this experiment, but the possibility to do so affects the statistics of the outcomes even in the standard setup. In quantum physics one cannot, in principle, ever measure or make predictions concerning the value of complementary variables simultaneously.

These differences require an adjustment of the concept of complementarity in applying it in psychology vs. quantum theory. Consider, for example, this claim, used to illustrate the role of complementarity in psychology, by the Clinton-Gore poll, just mentioned: “Once we obtain a measurement on say, Clinton, that decision can create a definite position for Clinton, but then the opinion regarding Gore must be uncertain” [21] (p. 11). Uncertain for whom? The opinion of the subject regarding Gore is only uncertain for an outside agent, but it may be certain for the subject, thus contrary to what this statement appears to suggest. An outside agent can never be certain what the subject thinks in general at any point, even if the subject provides as much information as possible concerning the context of this thinking. This is a crucial feature of the situation and of all human interactions, a feature discussed in detail below. In quantum physics, one only deals with the view of the agent, once the measurement is performed, which is part of Bohr’s complementarity in physics. There are psychological phenomena, such as those of the so-called bistable perception, sometimes associated with complementarity, in dealing with such objects such as the Necker cube or the Rubin vase, which lead to spontaneous alternatives between mutually exclusive perceptual states (e.g., [40]). However, in such cases, we do not and cannot makes a conscious decision concerning in which way we prefer to see. This is not complementarity in Bohr’s sense of it as a physical concept, which could only apply to human *decisions* (absent in bystable perception), still requiring suitable adjustments in considering human thinking, rather than quantum objects in physics. E. Rubin was a friend of Bohr, and some argue that his ideas influenced Bohr’s thinking about complementarity. That may be. However, this does not change the fact that Bohr’s concept as such is a different concept (from that of the mutual exclusivity in bistable perception), defined by all its aspects, considered above.

Even more significant is that, in the Clinton-Gore experiment, the statistics of the outcomes are at least in part defined by classically causal relationships between the two answers, “measurements”, in a given sequence, for well-established psychological reasons of, roughly, a guilt or, as it were, innocence by association. One can expect these answers and their (rough) statistics in advance because of these causal relationships, rather than, as in quantum physics, only because of previously performed statistical experiments of the same kind. In quantum measurement there are no classically causal relationships, or at least, they are, if possible, much more difficult to establish. Our statistics may only be expected or confirmed, because they were, or could be, found in the same type of experiments. Once one measures one complementary variable (say, the momentum), no expectations at all are experimentally possible as concerns a future value of the other (the position) at any future point, and this is true even if one assumes classical causality, as in Bohmian mechanics. In other words, in quantum physics when one is asked a subsequent question, information carried over from the preceding question does not and, in principle cannot, influence the subsequent response, contrary to Z. Wang and J. Busemeyer’s claim [21] (p. 11). No information carried from an earlier position measurement has any relevance for any subsequent momentum measurement. On the other hand, the essential individuality, uniqueness, of human thinking and of each of its instances is in place just as it is in quantum physics and, hence, QI and QD postulates, fully apply. This individuality is a key aspect of the situation, an aspect missed by Wang and Busemeyer’s discussion of complementarity in either quantum physics or psychology.

The nature of S-decisions in such experiments, while in these cases, more containing, is essentially the same as any decision of thought. Imagine somebody walking, with a map, in an area of labyrinthine streets of a city with a great architecture (Venice is a handy example, with its canals complicating one’s navigation even with a map). One can plan a trajectory ahead, but because nice buildings on other streets one passes by, or for no apparent outside reasons, one changes one’s actual route, makes new plans, etc., sometimes consciously, sometimes unconsciously. It is possible, as Khrennikov contends, that the unconscious makes all such decisions first and then transmits them, in a manner of quantum measurement to consciousness, but consciousness could also make such decision on its own accord [34].

For a more dramatic case, consider a poet deciding between two or more words to use in a given line, a decision finalized by the consciousness but ultimately coming from the unconscious. John Keats, in one of his most famous letters first says “head”, and then crosses “head” and writes “heart” over it, speaking of “sharpening one’s vision into the [head] [crossed out] heart and nature of Man” (Letter to J. H. Reynolds, 3 May 1818, [41], v. 1, p. 281). This gives further evidence (and there is plenty of such evidence) that in some of his poems he was making and changing decisions concerning using either of these two words, or between heart and brain, brain and mind or brain and head. All these words occur, sometimes as each possibly selected against the other in each pair, in his poetry. Sometimes they may appear to be interchangeable, but one cannot be certain whether they were for Keats. One could not be certain even if he were still alive and one could ask him. In some cases, however, they are manifestly not interchangeable. Thus, in “Ode to a Nightingale”, he opens with “My heart aches and a drowsy numbness/pains my senses” (ll. 1–2; emphasis added). This cannot be replaced with “brain” or “mind”, although, in principle, “head” is possible, even if extremely unlikely. The brain does, however, appear a bit later—“the dull brain”, which aims but cannot stop poetic thought, as it flies toward the nightingale, “on the viewless wings of Poesy,/Though the dull brain perplexes and retards” (ll. 33–34). In addition to their poetic quality, these lines, as much of Keats’s poetry, are also a remarkable description of the complexity of human thought (both memory and imagination) and decisions, conscious and unconscious, our thinking makes, or wants to make, can make, cannot make and so forth. Our brain often “perplexes” and constantly “retards” us: it delays the implementation of our decisions and the fulfillment of our desires. Our bodies retard flights of our thoughts and desires, poetic or erotic. In another letter Keats speaks of innumerable compositions and decompositions which take place between the intellect and its thousand materials”, in this case “before it arrives at [the] perception of Beauty”, still a “trembling delicate snail-horn perception”, always in the eye or, as the case may be, the blindness of the beholder (Letter to B. R. Haydon, 8 April 1818, [41], v. 1, p. 265). Keats borrows this view from J. Hazlitt’s description of “a continual composition and decomposition of its elements, a fermentation of every particle in the whole mass”, a description in turn borrowed from J. Dalton’s atomic theory (cited in [41], v. 1, p. 265, note 6). This process may appear continuous but in fact, as discussed below, it may only appear so, while it may in fact be underlain by a discrete structure and “quantum jumps”, in accord with the QD postulate. T. S. Eliot, who read Keats’s letters (which were, of course, not his only source) similarly said, in the lines used as my title for this article:

Do I dare

Disturb the universe?

In a minute there is time

For decisions and revisions which a minute will reverse.

(“The Love Song of J. Alfred Prufrock”, 1915, ll. 45–48)

Eliot’s “Do I dare?” is itself a decision question, and he repeats it and “Do I dare or do I not dare?” in the poem several times. The poem was written in 1915 (although Eliot knew about the old quantum theory, including Bohr’s 1913 theory), so the question of disturbing an object by observation did not arrive in physics yet. However, in life it did. Even a second can reverse such decisions and revisions, while at the same time making each unique and unrepeatable, even though some of them may be effects of repetition or continuity. Keats or Eliot, including in these poems, made and reversed their decisions many times for many words, reflecting qualia, even though the structure of each poem organizes and thus, to some degree, controls these qualia. 

Our thinking and the representations it creates are more akin to the way particles or, in the present view, the corresponding representations in measurements, transforms into one another in QFT, before a measurement finalizes one of them or a set of them, new each time. This was a remarkable feature of high-energy quantum physics and QFT that emerged with Dirac’s discovery of the positron as, according to Heisenberg, “perhaps the biggest change of all the big changes in physics of our [20th] century” [42] (pp. 31–33). In one and the same experiment, say, in quantum electrodynamics, QED, after the initial preparation, say, of an electron, one finds in the next measurement in the corresponding region, not only an electron (or nothing), as in QM regimes, but also other particles: a positron, a photon, an electron–positron pair; that is, in RWR-type interpretations, the events or phenomena (observed in measuring instruments) that we associate with such entities. That was a momentous change of our view of matter, even as against QM, or the low-energy, nonrelativistic QFT, where these types of effects are not observed, although it brought significant changes from QM. QED predicts which among such events can occur, and with what probability or statistics. Once one moves to still higher energies, the panoply of possible outcomes becomes much greater. In the case of QED, we only have electrons, positrons and photons, single or paired; in QFT, depending how high the energy is, one can literally find any known and possibly as yet unknown elementary particle or combination. It is as if instead of moving objects and motions conceived on the model in classical physics, one encounters a continuous emergence and disappearance of particles, further complicated by the role of virtual particles, or something in nature which compels one to introduce this concept.

It is well known that M. Gell-Mann borrowed the term “quark” from Joyce’s *Finnegans Wake* [38] (p. 118). It is far less known that Joyce’s masterpiece (1939) was in turn influenced by quantum theory, conceivably by the discovery of antimatter, which was widely discussed at the time, just as the Higgs boson or black holes are now and was known to Joyce. As Joyce says in the novel, undoubtedly referring to his own project in the book: “I am working out a quantum theory about it for it is really most tantalizing state of affairs” [38] (p. 149). Just as particles do in high-energy quantum physics, in Joyce’s novel words transform into each other and new words are created, such as famously, “chaosmos”, equally applicable to his process or to quantum physics. It is a separate question how likely a QFT-like model, the richest of available mathematical models in physics as concerns its transformational structure, is to handle such a process, or in general how likely Q-like models, as mathematical models, are likely to handle human thinking and decision making, or human experience, perhaps quantum-like in character, such as thinking that led Joyce to what became a representation known as *Finnegans Wake* or, again, Keats to what became, as representations, his poems. On the other hand, such models, even QM-like (in view of the role of entanglement that multiplies quantum states) and especially, QFT-like ones, are far more likely to work, at least partially, than c-models, because even in simpler processes our thinking and the interplay of consciousness and the unconscious within it can produce more transforming decisions or, in the first place, qualia than any classical-like model can handle. More importantly, even though Q-like models may not ultimately be able to capture or predict the richness of human experiences, they give us a new way of thinking about this experience.

I would like—A decision? A choice?—still to close this, literary, part of my discussion with Bohr, who once said “even in written papers, where we have the possibility of reconsidering every word, the question whether to let it stand or change it demands for its answer a final decision essentially equivalent to improvisation” [12] (v. 2, p. 79). Bohr, famous for his tendency of endlessly reconsidering every word of his papers, including, one surmises, in writing this very sentence, undoubtedly thought here of his own practice. However, the point is general and implies an essential individuality, uniqueness of our decisions, such as my own, in writing this very sentence (which I revised a few times even at the last moment) akin to that of art, to which Bohr refers in his appeal to improvisation.

What is, then, shared in quantum physics and Q-models of human thinking is the irreducible individuality, uniqueness of each experiment, in accord with the QI postulate, an irreducible individuality of each answer that either nature (in its quantum aspects) or the human mind provides. It is defined by the uniqueness of the state of either nature or mind at any given point in time, coupled to the ultimate discreteness of each phenomenon relative to any other, captured, by the QD postulate, even though our conscious perception of these events may appear as a continuous representational flow. I shall further comment on this last aspect of the situation below. How nature and mind, thinking, ultimately works may be unknowable or even inconceivable, unthinkable. Freud’s German word for the unconscious was *das Unbewusste*, originally used by F. Schelling in the 18th century, the unknowable, even to ourselves, in contrast to our conscious thought, unknowable to others as well. The unthinkable, *das Undenkbare*? Perhaps. Some Germans poets, Schelling’s contemporaries, such as F. Hölderlin, or again, Keats in England thought that this might be the case [43].

The uniqueness of each inner experience and representation, and their discreteness, here represented in QI and QD postulates, have been emphasized by D’Ariano and Faggin in their quantum-informational analysis or indeed theory of consciousness [44]. One is even tempted not to speak of “Q-like” because their theory may be seen as just quantum-informational. I want to now consider their argument, even if only in a summary-like fashion, because to do justice to its subtle nature, would require a separate article. (The same qualification applies to my discussion of Khrennikov’s argument below.) Their starting point, common to recent discussions of consciousness, is Chalmers’s “hard problem:”

*In his book The Character of Consciousness [5] David Chalmers states what he calls the hard problem of consciousness, namely the issue of explaining our experience–*

*sensorial, bodily, mental and emotional, including any stream of thoughts. Chalmers contrasts the hard problem with the easy problems which, as it happens in all sciences, can be tackled in terms of a mechanistic approach that is useless for the problem of experience. Indeed, in all sciences we always seek explanations in terms of functioning, a concept that is entirely independent from the notion of experience. Chalmers writes:*

*Why is the performance of these functions accompanied by experience? …*

*Why does not all of this information processing go on “in the dark” free of any inner feel?...*

*There is an explanation gap between the function and the experience.*

*An effective paradigm for comprehending the conceptual gap between “experience” and “functioning” is that of [a] zombie, which is behaviourally indistinguishable from a conscious being, nevertheless has no inner experience.*

[44] (p. 2)

It may be observed in this connection (while still giving Chalmers his due) that H. Weyl, in his classic book, *Space Time Matter*, in effect posed the hard problem, if, as it were, by way of a reversal, in order to put it aside (hence I say “in effect”). To be sure, there are other precursors. Weyl’s argument, however, has an additional relevance and appeal because it deals with physics, even if not quantum physics, which is only mentioned in passing in the book, originally published before QM. It also brings in philosophy, which is common for Weyl, but uncommon in technical works in physics, and Weyl’s books is as mathematically technical as any. Weyl argued that while physics, as a mathematical-experimental science, must, start with the experience of consciousness, as there is indeed no other point to start, it must ultimately leave this experience behind. He says, also speaking of “qualities”, which are in effect qualia, and using “green” and “leaf” (thinking perhaps of a green leaf) as his examples, thus also, fittingly, giving our visual experience (which is neurologically dominant in our brains) a special emphasis:

*It is easily seen that such a quality as “green” has an existence only as the correlate of the sensation “green” associated with an object given by perception, but that it is meaningless to attach it as a thing in itself to material things existing in themselves. This recognition of the subjectivity of the qualities of sense is found in Galilei (and also in Descartes and Hobbes) in a form closely related to the principle underlying the constructive mathematical method of our modern physics which repudiates “qualities”. According to this principle, colours are “really” vibrations of the æther, i.e., motions. In the field of philosophy Kant was the first to take the next decisive step towards the point of view that not only the qualities revealed by the senses, but also space and spatial characteristics have no objective significance in the absolute sense; in other words, that space, too, is only a form of our perception. In the realm of physics it is perhaps only the theory of relativity which has made it quite clear that the two essences, space and time, entering into our intuition have no place in the world constructed by mathematical physics. Colours are thus “really” not even æther-vibrations, but merely a series of values of mathematical functions in which occur four independent parameters corresponding to the three dimensions of space, and the one of time. …*

*One and the same leaf seems to have such and such a size or to be coloured in such and such a way, according to my position and the conditions of illumination. Neither of these modes of appearance can claim to present the leaf just as it is “in itself”. Furthermore, in every perception there is, without doubt, involved the thesis of reality of the object appearing in it; the latter is, indeed, a fixed and lasting element of the general thesis of reality of the world. When, however, we pass from the natural view to the philosophical attitude, meditating upon perception, we no longer subscribe to this thesis. We simply affirm that something real is “supposed” in it. The meaning of such a supposition now becomes the problem which must be solved from the data of consciousness. In addition, a justifiable ground for making it must be found. I do not by this in any way wish to imply that the view that the events of the world are a mere play of the consciousness produced by the ego, contains a higher degree of truth than naïve realism; on the contrary, we are only concerned in seeing clearly that the datum of consciousness is the starting-point at which we must place ourselves if we are to understand the absolute meaning as well as the right to the supposition of reality. … “Pure consciousness” is the seat of that which is philosophically a priori. On the other hand, a philosophic examination of the thesis of truth must and will lead to the conclusion that none of these acts of perception, memory, etc., which present experiences from which I seize reality, gives us a conclusive right to ascribe to the perceived object an existence and a constitution as perceived. This right can always in its turn be over-ridden by rights founded on other perceptions, etc.*

[45] (pp. 4–5)

It follows, then, that at least classical physics or relativity, by definition do not, and indeed, cannot, address the hard problem, as Weyl’s further elaboration on this passage makes even clearer [45] (pp. 3–5). Nor, as Weyl’s argument suggests as well, can those forms of philosophy that philosophically ground classical physics or relativity, including as realist theories, but especially insofar as they exclude the experience as the experience of qualia. Accordingly, the approach to the hard problem must be grounded otherwise. It is true that Weyl is only concerned with physics, as something that does not deal with the experience of consciousness and thus the hard problem, which he, accordingly, need not deal with. My point is only that he, remarkably, identified the hard problem (even if without mentioning it as such) in 1918, when his book was first published. Quantum theory may provide a suitable starting point to the hard problem because it essentially involves at least the decision of consciousness and thus human thinking, even if not qualia, but only as a starting point. One trajectory from this starting point could then proceed first to quantum information theory and then to a Q-like theory based in the quantum-informational approach, thus, to a quantum-informational-like theory of human thinking. This is the trajectory followed by D’Ariano and Faggin. According to them:

*There are currently two main lines of response to the hard problem: (1) the Physicalist view–with consciousness “emergent from a functioning”, such as some biological property of life …; (2) the Panpsychist view–with consciousness as a fundamental feature of the world that all entities have. What is proposed here is:*

*Panpsychism with consciousness as a fundamental feature of “information”, and physics supervening on information.*

[44] (p. 2)

D’Ariano and Faggin do assume, however, that “a nonreductive theory of experience will specify basic principles that tell us how basic experience depends on physical features of the world” [44] (p. 4). These theories do to not deal with the evolutionary biological emergence of thinking and consciousness, although this emergence is often used to support the physicalist view. It might be observed that we do not know if zombies, that is, zombies that possess all aspects of manifested human behavior actually exist, although Chalmers appears to think that they might. Related questions arise in considering the digital AI, at least as concerns those “behaviors” of computers that we associate with the effects of thinking, the other side of this problematic is the question whether computers can actually think or have experience, for example and in particular, the visual experience, in the way humans do, a question debated equally intensely. Without entering this debate, I would note, that, acknowledging many tremendous achievements in this field, one should, nevertheless, not confuse, as it sometimes done, clever programming with thinking. One should not forget either that “information”, too, is a human word, just as reality is, as Bohr reminded us [46] (p. 234). Effective as it may be, information is only a way, one way among others, in which we see the world, including quantum phenomena. There is a subtle but important difference in these two statements: “the Panpsychist view–with consciousness as a fundamental feature of the world that all entities have” and “Panpsychism with consciousness as a fundamental feature of ‘information’, and physics supervening on information”. The second statement need not imply, as is assumed in some panpsychist views, that all entities in the world have consciousness, views that I am disinclined to entertain and, in any case, put aside. The sentence may be read as a human view of the world, moreover, in its mathematical and thus still idealized aspects, which as I suggested above, may be limited as concerns how much of our thinking it can handle. As I argue here, however, it changes how we think about thinking.

D’Ariano and Faggin’s theory is based in discrete ontology or, more accurately, the ontology of discrete, each time, unique events of experience, the experience assumed to be “ontic” and quantum in character, while representations to our mind, as effects of this experience, effects assumed to be classical. In the present terms, experience would be ontological, insofar as it is represented by the mathematical formalism of quantum theory, as it is D’Ariano and Faggin. In discussing their argument, however, I shall use ontic as an adjective and ontology as a noun. My emphasis reflects the distinction used in in this article, which distinguished thinking (I did not primarily refer to experience), as an activity, akin to the workings of physical reality, and representations as effects of this activity, akin to those of quantum phenomena. Both types of effects are classical or classical-like, and thus representable, in nature, while neither activity may be representable or even conceivable, although it is assumed to be representable in the case of thinking in D’Ariano and Faggin’s theory. “Representation” in this sense follows Kant’s use of “*die Darstellung*”, which may be translated as a mental entity put forward in a representation by human thinking, thinking that, in its ultimate nature, may not be ultimately representable or knowable, although it is still assumed to be conceivable, thinkable by Kant, as against the strong RWR view, or in this case, because with we deal with mental entities, strong IWI (ideality-without-idealism) view. I shall return to this point in closing. In D’Ariano and Faggin’s argument, in contrast to the ontic nature of experience, any informational assessment of this experience or (classical) representations it creates is epistemic and generally mixed. This distinction is grounded in one of their principles, also assumed in this article, in accord with the QI principle (manifested in the QI postulate), as well as by Chalmers. D’Ariano and Faggin call it “Privacy principle:” “Experience is not sharable, even in principle” [44] (p. 5). D’Ariano and Faggin associate this principle with “the internally experienced quantum state”:

*We … argue that the internally experienced quantum state, since it corresponds* *to a definite experience–not to a random choice–must be pure, and we call it ontic.* *This should be distinguished from the state predictable from the outside (i.e.* *, the state describing the knowledge of the experience from the point of view of an external observer) which we call epistemic and is generally mixed. Purity of the ontic state requires an evolution that is purity preserving, namely a so-called atomic quantum operation. The latter is generally probabilistic, and its particular outcome is interpreted as the free will, which is unpredictable even in principle since quantum probability cannot be interpreted as lack of knowledge.*

[44] (p. 1)

Long-term memory, which may be seen as a vast “archive” of representations, from which each recollection extracts only a small item, is classical too. D’Ariano and Faggin state, as one of their main principles: “Memory is classical. Only the short-term buffer to collect each experience is quantum” [44] (p. 13). (This is strictly parallel to quantum physics, in which records of the past are always classical in terms of their physical representation, while predictions concerning future are always quantum-theoretical.) D’Ariano and Faggin do not consider the unconscious. It need not follow, however, that, while we have no awareness of this experience (we only have awareness of its representational and thus classical effects), this experience is available to our unconscious representations. This experience might instead be seen as equally productive of both conscious and unconscious representations, with long term-memory archiving the unconscious ones, while still, as *experience* remaining beyond unconscious representations that it produces. The process of bringing such representations, as recollections, into consciousness, or conversely “storing” them in one way or another (such as forgetting or repression) in long term memory, would still be a form of quantum experience:

*Quantum state evolution accounts for a short-term buffer of experience and contains itself quantum-to-classical and classical-to-quantum information transfers. Long term memory, on the other hand, is classical and needs memorization and recall processes that are quantum-to-classical and classical-to-quantum, respectively. Such processes can take advantage of multiple copies of the experienced state re-prepared with “attention”, and therefore allowing for a better quality of classical storing. …*

*The infocomplete measurement (or the random observables seen before) is better suited to extract classical information from the quantum buffer for long-term memory, since is does not privilege a particular observable. It is also likely that the conscious act of memori[z]ing an experience may be achieved by actually repreparing the ontic state multiple times in the quantum buffer, and performing the infocomplete test multiple times, thus with the possibility of memori[z]ing a (possibly partial) tomography of the state. … We have seen that an infocomplete measurement is needed to (approximately) store an experience in the classical long-term memory. By definition, the recovery of an experience requires a reproduction of it, meaning that the corresponding ontic state is (approximately) reprepared from the classical stored data. The memory, being classical, will be read without disturbance, thus left available to following recollections. … A possible benchmark for the memory store-and-recall process is the maximal fidelity achievable in principle with a measure-and-reprepare scheme that optimizes the fidelity between the experienced state and the recalled one.*

[44] (p. 15)

There are also effects of temporal continuity, appearing in view of small interval and small fluctuation between events, but only effects, because the ultimate ontology of events and even representation is still discrete, and, again, each time unique. One can illustrate this situation by returning to quantum theory, specifically to Heisenberg’s discovery of the uncertainty relations, based on the uniqueness of each quantum phenomena and their discreteness relative to each other (QI and QD principles) [47]. In his thinking leading to his discovery of the uncertainty relations, Heisenberg considered the visible and apparent continuous trajectory of an electron in a cloud chamber. He realized that the position of the electron was only known and the trajectory appears continuous because the water droplets, consisting of millions of atoms and thus much larger that the electron (which must be idealized as a point-like), condensed into this apparent continuity around the discrete events the traces of which spread through water. If one could zoom in on what actually happens, one would see this discreteness of the underlying phenomena and realize that the position and the momentum of the electron could not be known exactly, or indeed even defined simultaneously. In both Bohr’s and the present view, such variables could only be attributed to what is observed, classically, in the cloud chamber and not to the electron itself. It is clear that the continuity of quantum phenomena can only be apparent, underlain by the discreteness and uniqueness of each, in accord with the QD and the QI principles. On the other hand, the unconscious may not always be producing small fluctuation effects, as shown by our dreams, which scamble our memories and our daily (waking) experiential thinking and logic, or some long-term memory effects, which contain discontinuities, for example, by discontinuously switching us to earlier moments in time. 

Importantly, while still mathematically represented by a Hilbert-space formalism, D’Ariano and Faggin’s ontic evolution of experiences is not the unitary evolution of von Neumann but a different type of evolution, a pure, technically “atomic” evolution, which dictates the pure and ultimately discrete nature of each state of this evolution [44] (p. 9) [31]. This alternative mathematics grounds both the discreteness and uniqueness of each experience and representation, rather than represents the situation in terms of a continuous unitary evolution into which discreteness and uniqueness are brought, as interruptions, by measurement. D’Ariano and Faggin argue that the free will is only possible under their ontological and mathematical assumptions, while not possible under those of von Neumann. They also argue that at each point the free will is defined by the interconnected composition of multiple elements, each brought in its own discrete sequence of experiences, with quantum entanglement organizing qualia [44] (pp. 7–9). The necessary multiplicity of these ontic quantum experiences and classical representations they create could, D’Ariano and Faggin further argue, only be enabled by a quantum, and not classical, informational process, because of enormous resources of potential information (possible bits) already encoded in each qubit, and, again, the role of entanglement in creating and organizing ever greater multiplicities of qualia.

D’Ariano and Faggin’s scheme accords with the argument given earlier concerning the double structure of measuring instruments as both classical (in their observable parts) and quantum (in those parts of them through which they interact with quantum objects, or in the present view, the ultimate nature of physical reality responsible for both quantum objects and quantum phenomena at the time of measurement). As quantum or Q-like, the experience of consciousness in their scheme would be parallel to this quantum interaction, while what is observed in measuring instruments is a classical representation, which is an effect of this interaction. The relationships between D’Ariano and Faggin’s ontic and, thus, in present terms, conceptually ontological, view of experience, as defined by pure ontic states, and the RWR/IWI view is a more complex matter, which would require a separate discussion. A few brief comments could, however, be made here.

First, in RWR-type interpretations of quantum phenomena and QM, such as that of Bohr or the one adopted here, all quantum states (pure or mixed) in the sense of quantum-mechanical formalism are assumed only to be part of the mathematical machinery enabling or expectation-catalogs concerning what is, classically, observed, in quantum experiments. This view would be in conflict with any ontology of the physical reality responsible for quantum phenomena, for example, von Neumann’s unitary ontology. Von Neumann’s ontology, referred to by D’Ariano in the article, cited earlier, critiquing it, “as the ontological dogma”, grounds most ontological views in quantum physics [31]. While the difference between D’Ariano and Faggin’s view and RWR/IWI-type interpretations of thinking would still obtain, the mental nature of their ontology does change the situation. This is because the assumption of a mental ontology, that is, a representable character of mental reality (D’Ariano and Faggin only claim that their pure ontic states *represent* this reality rather than are this reality) is, philosophically, very different from assuming an ontology of physical reality, an assumption that defines scientific realism in classical physics, relativity and quantum theory. I would still prefer seeing the ultimate nature of mental reality in RWR/IWI terms (e.g., [48,49]), in part building on and given a more radical form to Kant’s view that things-in-themselves (vs. phenomena which are representations that our mind forms) may also be mental [50]. This tells us that our mind may be not be able to represent or to know itself, a point to which I return in closing. However, I do agree with D’Ariano and Faggin’s view that assuming a mental ontology is very different from assuming a physical ontology. The reason is that, if I can restate in this way R. Descartes’s ergo cogito sum, we only experience the reality of our experience and only construct the world that we perceive through this experience (to which D’Ariano and Faggin’s theory gives a Q-like ontology). How this experience is ultimately possible for us remains a question. This question may, however, only be posed here and not answered, beyond the fact, assumed here (without assuming a physicalist approach to thinking), that this possibility is still a product of our neurological machinery that emerges in our evolutionary history. 

As I said, I also put aside the question of free well, which is central to and is given a subtle quantum-informational treatment in D’Ariano and Faggin’s article, entitled, “Hard problem and free will: an information-theoretical approach”. I would, however, like to cite their summation of their argument:

*The outcome [of a quantum ontic transformation]* *Ft is a classical output, and we identify it with the free will of the experiencing system. It is a probabilistic outcome that depends on the previous history of qualia of the system. Its kind of randomness is quantum, which means that it cannot be interpreted as lack of knowledge, and, as such, it is free. Notice that both mathematically and literally the free will is the outcome of a transformation that corresponds to a change of experience of the observer/agent. The information conversion from quantum to classical can also take into account a stage of “knowledge of the will” corresponding to “intention/purpose”, namely “understanding” of which action is taken.*

[44] (p. 10)

The question here is, again: How free the free will ever is, given the exterior and interior, such as unconscious, reasons always affecting it? This question compelled me to put aside the category of free will, at least as concerns our decision making, even though in a certain sense, any representation that appears to our mind is a decision of our thought, possibly defined by our thinking experience as quantum in D’Ariano and Faggin’s sense. They are, however, correct insofar as our free will is interior-ontic, “that depends on the previous history of qualia of the system. Its kind of randomness is quantum, which means that it cannot be interpreted as lack of knowledge [on the part of the exterior observer/agent], and, as such, it is free”. Our ontic states of experience are free (or as concerns an outside agent, “objective”) insofar as, while an exterior agent can control, for example, the question asked, the agent cannot control the answer received, which has fundamental randomness. The case is analogous to quantum experiments. One can control the preparation of our experiment, but one cannot control the outcome, in the way one does, at least ideally, in classical physics. D’Ariano and Faggin’s formalism captures this situation in a new way by virtue of their nonunitary ontology.

I have only offered (and I promised no more) an outline of their argument, focusing on the points most relevant to the argument of the present article. These points are the uniqueness of each experience or event and the discreteness of all events and representations they give rise to, unfolding, as discrete, events in multiple sequences, represented here in terms of the QI and the QD postulate, and the ultimately unrepresentable nature of these experiences or even these (classical) representations by an outside agent, such as another human subject. As D’Ariano and Faggin acknowledge, their argument does not prove that the nature or structure of consciousness is quantum, but it suggests that assuming it to be such is a better Bayesian bet than assuming it to be classical. One cannot prove such theory, but one can bet on it, even if without certainty at this point. This is also true about QM or QFT in physics, although we have much better experimental data (than in the case of human thinking) to support betting on it. 

I shall now consider Khrennikov’s argument, which brings the unconscious into Q-like theorizing of human thinking, by understanding consciousness in terms of measurements performed on the unconscious, measurements viewed as decisions by consciousness. It is not entirely clear whether, in contrast with D’Ariano and Faggin in the case of their nonunitary evolution (expressly opposed by them to von Neumann’s unitary evolution), Khrennikov assumes a mental ontology based on von-Neumann unitary evolution. One might plausibly conjecture, as I shall do here, that he does, given his use of Ozawa’s argument concerning quantum measurement, which assumes von Neumann’s unitary ontology [22,32,33]. I shall not, however, exclude the possibility that Khrennikov leaves this aspect of the situation open, and shall indicate why he might do so as I proceed. Either way, Khrennikov sees decision making or, it appears, any given instance of conscious thinking, thus seen as a decision, in terms of consciousness (C) making a measurement on the unconscious (UC) [34,51]. Khrennikov does not distinguish experience and representation in the way I do in affinity with D’Ariano and Faggin’s view. A human subject, at least part of it, is thus also an interior measuring instrument which produces each such outcome. It is an important idea and I shall adopt it here, as concerns the role of the presence of something in consciousness as a form of measurement in human thinking, and will try to build on it by suggesting that human thinking as experience, Q-like in nature, creates by performing something akin to a quantum measurement of both conscious and unconscious representation, which are, as representations, classical. The present understanding of quantum measurement, following Bohr, as outlined above is crucial here (hence my emphasis) and is, I would contend, consistent with Khrennikov’s argument. To reprise this understanding, a quantum measurement does not measure any property of the reality responsible of what is observed, which it would possess before or during the act of observation, which establishes quantum phenomena by an interaction between the instrument and the quantum object. In quantum physics what is so observed can be measured classically, just as one measures what is observed in classical physics, where, however, what is so measured could be associated with the object itself considered for all practical purposes. In the case of consciousness, the outcomes of our measurements upon the unconscious are manifested to our consciousness in classical concepts, through which these outcomes can then be made manifested to an outside observer.

The C-US system is, thus, modelled by Khrennikov on the MI-O, (measuring) instrument-object, system, as used in “the indirect quantum measurement”, a more general concept that follows Bohr but instantiated in terms of Ozawa’s mathematized scheme of quantum measurement, mentioned earlier [22,32,33]. This view is related, which is not to say strictly conforms, to the tripartite scheme introduced by the present article—quantum phenomena, the ultimate RWR-type reality responsible for them, and quantum objects, defined, along with quantum phenomena, at the time of measurement—under the assumption of the movable cut between what is considered as the object and the instrument in each measurement. As explained above, D’Ariano and Faggin’s view is also consistent with this tripartite scheme, insofar as human experience in their sense is parallel to the Q-like reality responsible for quantum phenomena observed and classical representations, as an effect of the experience, are parallel to quantum phenomena. There is, however, no appeal on their part to either measurement or the unconscious. On the other hand, if “quantum state evolution [which is, in this case nonunitary and atomic] accounts for a short-term buffer of experience and contains itself quantum-to-classical and classical-to-quantum information transfers”, quantum-to-classical information transfers may be seen as measurements and, as I shall suggest, these transfers themselves may be extended to the unconscious [44] (p. 15).

The part of this tripartite scheme most relevant to Khrennikov’s approach is the double structure of measuring instruments as both classical in their observable parts and quantum in those parts of them through which they interact with quantum objects, or in the present view, with the ultimate reality responsible for both quantum objects and quantum phenomena at the time of measurement. This interaction then gives rise to the quantum object, as well as the phenomenon, considered at the time of measurement. This view is especially relevant in the present context, because, as noted earlier, there are no analogues of quantum objects in human thinking, in which every instant of thought creates a new “object” of experience, responsible for the corresponding representation. This type of cut could be applied to human thinking and decision making, as regards the assumption or the decision by an outside agent concerning how the human subject considered made the decision, say, in response to a given question. That is, different agents can make different assessment as concerns what is conscious and what is unconscious in the subject’s decision. Khrennikov’s “scheme of indirect measurements”, presented by him via [22,32,33], cited above and Khrennikov’s main source, corresponds to the double (classical and quantum) structure of measuring instruments and measurement, applicable even if this structure in interpreted in realist terms, as it is by Ozawa and possibly by Khrennikov. Ozawa manifestly assumes von Neumann’s unitary evolution and his view of measurement, which evolution is, as noted, seen in realist terms by von-Neumann. This would, again, suggest that Khrennikov assumes von Neumann’s scheme as well. As belonging to the RWR-type, Bohr’s and the present interpretation do not assume the unitary-evolution ontology and especially von Neumann’s view of measurement, or any physical ontology, including that based on D’Ariano and Faggin’s nonunitary evolution. As discussed above, however, because this evolution represents a mental, rather than physical, reality, is a mental ontology, the situation is different, as it is also in the case of Khrennikov, whether he ultimately assumes, as I surmise here, the unitary-evolution scheme or not.

It might be helpful to reprise Ozawa’s scheme of measurement (my notations, used earlier, are slightly different from Khrennikov). One considers a compound quantum system, QO + QI, consisting of the quantum object under investigation, QO and the quantum part, QI, of the instrument I (QO + QI), which is isolated during the (short) time interval when the quantum interaction in question takes place. The rest of the instrument, I, performs a pointer measurement, on QI, after the interaction has taken place. In realist views, such as that of M. Ozawa, the evolution of the QO + QI, the unitary evolution operator, $U\left(t\right)\;=e^{-i\Delta tH}$, where *H = H_QO_ + H_QI_ + H_QOQI_* is the Hamiltonian representing the internal behavior of the subsystems involved and *H_QOQI_* the interaction between them. According to Khrennikov, applying this scheme to the C-UC system of human thinking:

*The scheme of indirect measurements … was created in quantum physics and applied successfully to [a] plenty of important problems. Our aim is to adapt it to cognition. The main question is about cognitive analogues of the system S and the measurement apparatus, M. We suggest using the framework developed in paper [50] for quantum-like modeling of the Helmholtz sensation-perception theory [32,33]. This scheme can be extended to a general scheme of unconscious-conscious interaction in the process of decision making.*

*The measured system S is a sensation (or generally any state of the unconscious mind). Consciousness as the measurement apparatus to the unconscious interacts with sensations to make the decisions (to generate outcomes of measurements). Consciousness, of course, is not concerned just with a single probe. It is a large environment with many probes interacting with the unconscious. There should be a rule of transformation from a sensation to a conscious decision. This is unitary transformation and measurement of the meter in the probe. The operational description neglects neurophysiological and electrochemical structures of interaction. The unconscious is a black box that is mathematically described by the state of sensation space (=the unconscious state), so that the unconscious state probabilistically determines the decision (by interaction with consciousness), then the unconscious state is changed according to the previous unconscious state and the decision made. Thus, each probe is described by a quantum instrument. Instruments are probe dependent.*

*For the question-measurements, the question A is transferred into the unconscious, where it plays the role of a sensation…, so to say a high mental level sensation. Then, interaction described by the unitary operator U generates a new state of the compound system-the unconscious-conscious. In addition, consciousness performs the final “pointer reading”, the measurement of the meter observable. Pointer reading can be treated as generation of a perception, a high mental level perception.*

[34] (p. 8)

It follows that: (a) as an outcome of measurement, conscious thinking or, in my preferred terms, representation is classical in nature; and, at least by implication, (b) each instance of thinking, registered by consciousness is unique and is discrete in relation to any other, thus, on both counts, in accord with a view, defined by the QI and QD postulates, adopted in this article. ($U\left(t\right)\;=e^{-i\Delta tH}$ is the “decision time” and it is random, which makes the impression of free will, a subject, again, put aside here, beyond the limited considerations given above. While the brain is assumed by Khrennikov to be a black box physically, it is not entirely clear that the unconscious as such is a mental black box and in what sense, if Khrennikov assumes a Q-like ontology of the unconscious, whether as represented by von Neumann’s unitary revolution or otherwise. One might read this appeal to a black box as an indication that Khrennikov does not assume an ontology (of whatever type) representing the mental reality considered. Given, however, Khrennikov’s overall argument, as just discussed, especially, again, his use of Ozawa’s measurement scheme, I am inclined to assume that he does. Notably, and given the mental ontology they assume, logically, D’Ariano and Faggin do not speak of the experience of consciousness as a black box, although, one might surmise, they, too, would see the brain as a black box physically. 

Now, while some instances of conscious thinking may and even must be seen as outcomes of measurement performed on the unconscious, it seems doubtful, given the richness of our conscious experience and thinking, that *all* conscious thinking or, at least, representations are the outcomes of such measurements upon the unconscious thinking. If one adopts this view, conscious thinking is, in a way, not really thinking, but a machinery, a mental technology, that brings representations created by the unconscious into their presence in consciousness. This assumption is not impossible, and I would argue, is at least partially true, insofar as consciousness has this function, and it may well be that the dominant and most essential thinking activity is unconscious, as Freud appears to have thought, without, however, denying thinking as such to consciousness either. On the other hand, Freud never delivered on his promise of offering a theory of consciousness, which he even saw as more enigmatic than the unconscious, extensively theorized by him. It is possible, however, while retaining the idea that some form of measurement is part of thinking, measurement that thinking performs on itself, and Freud’s emphasis on the unconscious, to take a somewhat different view of the relationships between consciousness and the unconscious than only seeing consciousness as performing measurements on the unconscious. Freud and his emphasis on the unconscious are actually mentioned by Khrennikov, if hesitantly, vs. W. James, as a safer “alternative”: “We can appeal to the authority of James … (appealing to Freud … might generate a negative reaction)” [34] (p. 9). Here, in closing, I will risk a more sustained appeal to Freud, in part following [52] (pp. 11–12), even if without fully subscribing to Freud’s views either, in part given their ultimately realist nature, vs. the RWR/IWI view assumed here. In a way, the present view is closer to Kant, even if, as explained, more radical than that of Kant as well. James is a separate matter, which will have to be put aside here. In any event, it is Freud or earlier F. Nietzsche who are primarily responsible for bringing the question of the unconscious to the center stage of modern philosophy and psychology.

## 5. Conclusions: From Kant to Freud, and Beyond, with Quantum-Like Thinking

Freud adopts Kant’s view based on the difference between phenomena, defined by appearances to, or representations [*die Darstellungen*] in, our mind, which are available to our knowledge, vs. noumena or things-in-themselves as they actually exist, which are beyond our knowledge, but not necessarily beyond thought, as they would be in the strong RWR view, adopted here. Freud builds on Kant’s important and sometimes overlooked contention that things-in-themselves may also be mental. In Freud this noumenal domain is the unconscious. While, as I said, Freud’s German term for the unconscious is *das Unbewusste*, originally introduced by Schlegel (in part in response to Kant), Freud was more optimistic than Kant as concerns a possible knowledge of the unconscious. In his 1915 “The Unconscious”, Freud says, perhaps also thinking of quantum theory then, such as Bohr’s 1913 theory: “The mental, like the physical, is not necessarily in reality what it appears to us to be. It is, however, satisfactory to find that the correction of inner perception does not present difficulty so great as outer perception—that the inner object is less hard to discern truly than in the outside word” [7] (p. 121).

Freud might have been overconfident as concerns the capacity of his psychanalytic theory to discern the ultimate nature of this “inner object”, that is, the ultimate character of mental reality, for example, famously, in terms of the Oedipal structure of the unconscious. Freud, as noted from the outset of this article, decoupled this mental reality from the physical workings of the brain and thus was not a physicalist. In any event, I want, by contrast, to suggest, on RWR lines, that we may not have such a capacity. One can still maintain the difference between this ultimate, RWR-type, unconscious reality and its classical-like effects, effects that can, in this view, be either conscious and unconscious classical “representations” [*die Darstellungen*] or qualia, akin to effects of measurement manifested in quantum phenomena in quantum physics, without being representations of this reality itself, not possible in the RWR view. Such conscious and unconscious representations or qualia may, for example, be seen as effects of *experience* in D’Ariano and Faggin’s sense of experience as a quantum-informational mental entity. In their view, at least in their argument in their article considered here, this experience is the experience of consciousness, given by them an ontology and thus a representation, a representation is not classical and, hence, is different from classical representational effects this experience gives rise to. One might argue, however, that it is possible to assume (a) that our experience is ultimately the Q-like experience of the unconscious, which creates both conscious and unconscious classical, C-like, representations or qualia as its effects, and (b) that this experience may not be given ontology and thus any representation or even conception, and hence, form an RWR-type reality. It is true that classical unconscious representations or qualia created, as effects, by this reality remain hidden and could only be manifested, indirectly, in conscious representations or qualia, for example, via memories or dreams, two main phenomena manifesting the unconscious for Freud. Dreams, which Freud called “the royal road to the unconscious”, pose further complexities to our understanding of experience and qualia, complexities that were one of the main reasons for Freud’s introduction of his psychoanalytic theory in the first place. His first book was *Interpretations of Dreams* [53]. These complexities also involve the role of desire (which, according to Freud, shapes our dreams) in human experience, which is separate subject put aside here. I shall only mention that we, in general, do not have a conscious experience of a dream, but only a conscious recollection of it, a form of a memory of something that we had never experienced consciously. For the moment, my suggestion that our experience is underlain and is shaped by the ultimate RWR-type and thus unrepresentable unconscious reality would make indirectly *manifested* phenomenal representations of hidden unconscious representations double effects of the Q-like ultimate experience and, as such, representations of representations—representations of unknown (but not entirely unknowable) representations. This, however, still allows for the overall scheme thus suggested, according to which our Q-like, RWR-type, unconscious experience creates both conscious and unconscious classical representations or qualia. At the same time, maintaining the parallel with quantum measurement in physics and, thus, retaining the measurement aspect of Khrennikov’s argument, both conscious and unconscious representations or qualia might be seen as classical effects arising in Q-like measurements. By the same token, if, as D’Ariano and Faggin argue, “quantum state evolution accounts for a short-term buffer of experience and contains itself quantum-to-classical and classical-to-quantum information transfers”, quantum-to-classical information transfers in their scheme may also be seen as measurements and extended to the unconscious [44] (p. 15).

But measurements of what? This is the question. This question is, however, asked keeping in mind that a measurement is understood here as a creation of phenomena through the interactions by one or another means (such as measuring instruments or consciousness) with the reality considered (such as matter or the unconscious), rather than measuring the preexisting properties of this reality. Perhaps, if we put aside the physicalist view, what is thus measured, measured without measuring (in the classical sense), are the ultimate mental things-in-themselves, which Kant already invoked nearly three centuries ago, or even a more radical ideality-without-idealism (IWI) form of mental reality [50]. Heisenberg eventually saw Kant’s things-in-themselves, if assumable at all in physics, as “mathematical structure[s]” [54] (p. 52). Heisenberg’s view implies a form of ontology, although in this case as representing the ultimate constitution of nature, apart from physical concepts, rather than the ultimate nature of human thinking, which is at stake in the present argument. I am not saying that Kant had envisioned what I suggest here, especially given that, if assumed as it is here, as a form of reality without realism, this mental reality is more radical than that of Kant’s things-in-themselves, only assumed by him to be beyond knowledge and not beyond conception. This reality, now as mental (rather than material) reality, is beyond thought altogether, beyond anything our thinking can conceive, because our thinking can only give rise to representations (“*die Darstellungen*”) or qualia that are classical in nature. This reality could not be given any representation or ontology, mathematical or other, and thus cannot be “measured” any more than material, physical reality responsible for quantum phenomena could be in the strong RWR view. Hence, as a form mental reality, this reality, while still as a reality without realism, becomes an ideality without idealism, IWI, where “idealism” is understood as the assumption of an entity, for example, mathematical as in Plato, that would represent or at least be a conception of this mental reality. The first “I” of IWI would then also be standing for the Ideality of the subject, its Identity and Individuality, the ultimate nature of which cannot be represented or even conceived of.

While the view just sketched may not solve the hard problem, it will further change what it could mean to “explain” thinking or consciousness (or the unconscious) or “solve” the hard problem, the meanings already changed by quantum theory and still further by quantum information theory, and hence by Q-like approaches to thinking, even ontological ones, such as that of D’Ariano and Faggin, or that of Khrennikov (assuming, again, that it is ultimately ontological), different as these two approaches are. The question “The measurements of what?” does not entirely disappear even if one assumes an ontological view of the experience of consciousness or the unconscious. For, while we may assume that we think, we still do not know or cannot be certain what thinking ultimately is, even if we represent it in this way, as D’Ariano and Faggin acknowledge. The view just outlined, as the IWI view of our mental reality, would add to this transformation of our thinking concerning the nature of thinking.

I am not contending that the IWI view is the only possible starting point for approaching human thinking and decision making. For one thing, my main argument, defined by the postulates adopted from quantum physics, especially QI and QD postulates, permits different Q-models and theories, and different interpretation of them, and hence, different way of dealing with human thinking and decision making, even within the Q-like paradigm. But this is only one reason. There have been quite a few questions in this article that were posed in order to be posed rather than to be answered, especially definitively answered, such as and in particular the question, asked via literature, of how much of human thinking could be handled even by Q-like mathematical theories or models, hugely more capacious than classical ones as they are. As I have argued here, however, these theories, or quantum theory in the first place, suggest different ways in which we can relate to the world, the world of nature and the world of thought, in their irreducible interaction. This article, including as concern the RWR view, now also as the IWI view of human experience and thinking, is, too, only a wager, a bet. One cannot be certain that quantum theory is true even in physics, let alone as a model for our Q-like theories of human experience and thinking. But one can bet on both, give them a chance in the auction of our theoretical life, in which each of us is a seller, a buyer and an auctioneer, all at once.

## Data Availability

Not applicable.
